# Acidic Environment Leads to ROS-Induced MAPK Signaling in Cancer Cells

**DOI:** 10.1371/journal.pone.0022445

**Published:** 2011-07-26

**Authors:** Anne Riemann, Bettina Schneider, Angelika Ihling, Martin Nowak, Christoph Sauvant, Oliver Thews, Michael Gekle

**Affiliations:** 1 Julius-Bernstein-Institut für Physiologie der Universität Halle-Wittenberg, Halle, Germany; 2 Klinik und Poliklinik für Anästhesie, Universität Halle-Wittenberg, Halle, Germany; 3 Institut für Physiologie und Pathophysiologie, Universität Mainz, Mainz, Germany; Vanderbilt University Medical Center, United States of America

## Abstract

Tumor micromilieu often shows pronounced acidosis forcing cells to adapt their phenotype towards enhanced tumorigenesis induced by altered cellular signalling and transcriptional regulation. In the presents study mechanisms and potential consequences of the crosstalk between extra- and intracellular pH (pH_e_, pH_i_) and mitogen-activated-protein-kinases (ERK1/2, p38) was analyzed. Data were obtained mainly in AT1 R-3327 prostate carcinoma cells, but the principle importance was confirmed in 5 other cell types. Extracellular acidosis leads to a rapid and sustained decrease of pH_i_ in parallel to p38 phosphorylation in all cell types and to ERK1/2 phosphorylation in 3 of 6 cell types. Furthermore, p38 phosphorylation was elicited by sole intracellular lactacidosis at normal pH_e_. Inhibition of ERK1/2 phosphorylation during acidosis led to necrotic cell death. No evidence for the involvement of the kinases c-SRC, PKC, PKA, PI3K or EGFR nor changes in cell volume in acidosis-induced MAPK activation was obtained. However, our data reveal that acidosis enhances the formation of reactive oxygen species (ROS), probably originating from mitochondria, which subsequently trigger MAPK phosphorylation. Scavenging of ROS prevented acidosis-induced MAPK phosphorylation whereas addition of H_2_O_2_ enhanced it. Finally, acidosis increased phosphorylation of the transcription factor CREB via p38, leading to increased transcriptional activity of a CRE-reporter even 24 h after switching the cells back to a normal environmental milieu. Thus, an acidic tumor microenvironment can induce a longer lasting p38-CREB-medited change in the transcriptional program, which may maintain the altered phenotype even when the cells leave the tumor environment.

## Introduction

Two microenvironments can be distinguished with respect to solid tumors: (i) the tissue environment in which the tumor cells reside (pathological tissue environment) and (ii) the local environment created by the tumor cells (tumor microenvironment), that can generate a pathological tissue environment for neighboring cells. The pathological tissue environment supports tumor promotion and the tumor microenvironment supports tumor progression [1]–[4]. Tumor microenvironment is characterized by oxygen deficiency (hypoxia), as a consequence of structural and functional abnormalities of the vascular network [5], leading to inadequate perfusion of the solid tumor [5], [6]. In order to maintain the energy demand tumor cells switch their metabolism to glycolysis, resulting in increased glucose consumption and pronounced lactic acid production. This phenomenon can even occur in tumors when the oxygen supply is sufficient - known as the Warburg effect. Recently, evidence was presented demonstrating that splice isoform expression of pyruvate kinase is necessary for the altered metabolism which provides a selective advantage for tumor cells [7]. Together these features form a complex network and create a metabolic microenvironment, consisting of hypoxia, low glucose, high lactate concentrations and extracellular acidosis. pH values in the solid tumors are in the range of 6.5 to 6.8 [6]. This acidic environment is import for tumor promotion and progression.

It is well known that the metabolic microenvironment impacts tumor cell behavior. For example, the efficacy of radiation therapy, photodynamic therapy and chemotherapeutics is impaired by the tumor environment [8], [9]. Growth and migration characteristics as well as apoptosis sensitivity can be influenced, too. Thus, the phenotype of tumor cells - and therefore of the tumor itself - depends, in addition to the genetic determination, on the metabolic microenvironment. The “seed and soil„-hypothesis even postulates that after acquisition of all necessary cancerous genetic alterations only the formation of the tumor microenvironment allows tumor cells to grow [10].

For a detailed mechanistic understanding it is important to deconstruct this microenvironment and determine the effects of the different parameters individually in order to evaluate their contribution. Whereas there is ample literature on hypoxia, the importance of metabolic acidosis is less well investigated. Recently we showed that metabolic acidosis per se enhances chemoresistance in prostate tumor cells under normoxic and normoglycemic conditions [9], [11], indicating that acidosis is an important microenvironmental determinant for tumor phenotype alterations. This acidosis-induced stimulation of P-glycoprotein-dependent chemoresistance depends on MAP kinases, however it is unclear how the activation of these kinases by an extracellular pH-reduction occurs [9]. It may depend on intracellular changes of pH-homeostasis and its regulation in response to extracellular acidosis. Furthermore, there are several candidate signaling pathways that could link pH-changes to MAPK activation, e.g. the kinases PKA, PKB, PKC, c-Src or EGFR [12]. Therefore the aim of the present study was to examine (i) the pH-homeostasis of tumor cells during metabolic acidosis of the microenvironment, (ii) the mechanisms of ERK1/2 and p38 phosphorylation under these conditions and (iii) the possible relation between these two processes as well as the consequences of affecting these pathways.

## Materials and Methods

### Cell culture

The subline AT1 of the rat R-3327 Dunning prostate carcinoma was used as described before [9]. Cells were grown in RPMI medium supplemented with 10% fetal calf serum (FCS) at 37°C under a humidified 5% CO_2_ atmosphere and sub cultivated twice per week. LS513 cells (American Type Culture Collection, Rockville, MD, USA; CRL-2134) were grown under the same conditions as AT1 cells. OK cells (normal epithelial cells from renal proximal tubule of the opossum kidney) [13] and NCI-H358 cells (human bronchioalveolar carcinoma; American Type Culture Collection, Rockville, MD, USA; CRL-5807) were grown in DMEM medium supplemented with 10% FCS, MDCK-C7 cells (normal renal collecting duct epithelium cells of the canine) [14] in MEM medium supplemented with 10% FCS and CHO cells (immortalized ovary cells of the Chinese hamster) [15] in Ham's F-12 medium supplemented with 10% FCS. For all experiments, cells were grown in Petri dishes (western blot) or cover slips (measurement of pH_i_) and transferred to medium without additional FCS supplementation for 24 hours. Control cells were exposed to bicarbonate-HEPES buffered Ringer solution adjusted to pH 7.4. Intracellular acidosis was induced replacing 40 mM NaCl by 40 mM lactic acid and reduction of chloride by completely replacing NaCl by sodium gluconate (7.4 mM residual chloride). Extracellular acidosis (pH 6.6) was applied using bicarbonate-MES (morpholinoethanesulfonic acid) buffered Ringer solution pH-adjustment to 6.6. The buffer capacity (β) is 5.9 mmol/lxΔpH for the bicarbonate-HEPES buffered Ringer solution at pH 7.4 and 3.9 mmol/lxΔpH for the bicarbonate-MES buffered Ringer solution.

### Experimental setup

After serum depletion for 24 h, cells were incubated with one of the above mentioned buffers (1 ml) for up to 3 h. All inhibitors or activators used were added during this incubation period.

### Cytosolic pH

Cytosolic pH of single cells was determined using the pH-sensitive dye BCECF (2′,7′-bis-(2-carboxyethyl)-5-(and-6)-carboxyfluorescein, acetoxymethyl ester, Invitrogen, Paisley, UK) as described before [16], [17]. In brief, cells were incubated with Ringer solution containing 5 µM BCECF-AM for 15 min. Then, the cover slips were rinsed 2 times with superfusion solution to remove the excess of the dye and transferred to the stage of an inverted Axiovert 100 TV microscope (Zeiss, Oberkochen, Germany). The excitation wavelengths were 450 nm/490 nm, emitted light was measured through a bandpass-filter (515–565 nm). The data acquisition rate was one fluorescence intensity ratio every 10 s. After background subtraction, fluorescence intensity ratios were calculated. pH calibration was performed after each experiment by the nigericin (Sigma, St. Louis, USA) technique [18], [19] using a two-point calibration (pH 6.8 and 7.5). The calibration solutions contained 132 mM KCl and 1 mM CaCl_2_, 1 mM MgCl_2_, 10 mM HEPES and 10 µM nigericin.

### Cell volume

The effect of acidosis on cell volume was determined electronically with a Casy cell counter (Innovatis, Reutlingen, Germany). Therefore cells were incubated as mentioned above, detached by trypsinisation and cell volume and viability was measured after 10 min or 3 h in the respective Ringer solutions.

### Western blot

Western blotting was performed according to standard protocols. Cells were lysed (0.1% Triton X-100 in PBS, protease inhibitor cocktail, 37 mg/l sodium orthovanadate or 150 mM NaCl, 10 mM Tris pH 7.4, 1% Nonidet P-40, 0.1% SDS, 1% sodium deoxycholate, 0.1% Triton X-100, 1 mM EDTA, protease inhibitor cocktail, 184 mg/l sodium orthovanadate), cell protein determined by the BCA-methode (BC Assay Reagents from Uptima), separated by SDS-PAGE and transferred to a nitrocellulose membrane. Subsequently, membranes were incubated with antibodies specific for ERK1/2, p38, MKK3/6; phospho-ERK1/2, phospho-p38, phospho-MKK3/6, CREB and phospho-CREB (1∶1000, Cell Signalling). The bound primary antibody was visualized using horseradish peroxidase-conjugated secondary antibodies and the ECL system (Pierce/Thermo Fisher Scientific) with the Molecular Imager ChemiDoc XRS System (Biorad, Munich, Germany). Quantitative analysis was performed with Quantity One software (Biorad).

### CRE-SEAP reporter gene assay

Transactivation was assessed by the Mercury™ Pathway Profiling reporter gene assay system from Clontech Inc. using secretory alkaline phosphatase (SEAP) under the control of defined cis-regulatory response elements (CRE) as reporter, essentially as described earlier [20], [21]. In brief, the cells were transfected with a pCRE-SEAP constructs or empty vectors. SEAP-activity in the media was determined with the AttoPhos® System from Promega (Mannheim, Germany) and normalized to the transfection control (ß-galactosidase).

### LDH release

LDH activity in media and in cell lysates was measured using standard protocol [22] adapted to lower scale (200 µl) in a multiwell reader (Infinite, Tecan, Berlin).

### Caspase-3-activity

Cells were washed once with PBS buffer (4°C) and incubated with 100 µl cell lysis buffer (10 mM TRIS, 100 mM NaCl, 1 mM EDTA, 0.01% Triton X-100, pH 7.5) for 10 min on ice, harvested, and centrifuged at 16000g for 10 min at 4°C. 60 µl of the supernatant was incubated with 65 µl reaction buffer (20 mM PIPES, 4 mM EDTA, 0.2% CHAPS, 10 mM DTT, pH 7.4) containing 42 µM DEVD-AFC (final concentration) at 37°C, and fluorescence of the cleaved product, 7-amino-4-trifluoromethylcoumarin (AFC), was measured at 400 nm excitation and 505 nm emission wavelength using a multiwell counter (Infinite, Tecan, Berlin). Cleaved AFC was quantified by a calibration curve using known AFC concentrations. Protein content was determined with bicinchoninic acid assay (Interchim, Montluçon, France) using bovine serum albumin as standard.

### Determination of extracellular pH, glucose and lactate

pH was measured with a blood gas analyzer (ABL5, Radiometer, Copenhagen, Denmark). For determination of the concentration of glucose, the glucose (HK) assay kit from Sigma (G3293) was used (2 or 5 µl sample, respectively, plus 200 µl kit) according to the manufacturer's instructions. Lactate was determined using lactate reagent (Trinity) according to the manufacturer's instruction (10 µl sample plus 100 µl kit). Each experiment was performed at least in triplicates.

### ROS formation

Formation of reactive oxygen species (ROS) was assessed with the fluorescent dye DCFDA-AM (Molecular Probes, Leiden, Netherlands) which reacts with an increase in fluorescence in the presence of H_2_O_2_ once the ester bond has been cleaved by cellular esterases. Cells were seeded in 24-well-plates and incubated for 30 minutes with dye after the indicated treatments. Subsequently, fluorescence (excitation 485 nm; emission 535 nm) was measured using a multiwell counter (Infinite, Tecan, Berlin, Germany). Additionally blank values without cells and blank values without dye were determined and subtracted. The increase of fluorescence over the blank value expressed per mg protein was used as a measure for ROS formation.

### Determination of mitochondrial activity

In order to estimate mitochondrial activity, we determined the accumulation of rhodamine 123 after exposure to pH 7.4 or pH 6.6. After the 3 h exposure the cells were incubated for 20 min with 0.1 µM rhodamine 123 in HEPES-Ringer solution as well as in the presence of 20 µM CCCP or 2 µM nigericin [23], [24]. At the end the cells were lysed with 0.1% Triton X-100 and cellular fluorescence determined using a multiwell counter (Infinite, Tecan, Berlin). Mitochondrial uptake of rhodamine 123 is prevented in the presence of CCCP because Ψ_m_ collapses. By contrast, mitochondrial uptake of rhodamine 123 is maximal in the presence of nigericin, which collapses the pH gradient across the inner mitochondrial membrane. As a consequence the entire energy from the electron transport chain is transformed into Ψ_m_. Thus, uptake of rhodamine 123 in the presence of nigericin minus uptake of rhodamine 123 in the presence of CCCP represents a crude estimate of mitochondrial activity and eliminates any non-specific effects. Uptake of rhodamine 123 was calibrated using lysis buffer containing known concentrations of rhodamine 123.

### Materials

If not stated otherwise, chemicals were from Sigma-Aldrich, Munich, Germany.

### Data analysis

Data are presented as mean ± SEM. For all experiments, N equals the number of culture plates or cells used to perform the measurements if not quoted otherwise. All experiments were performed with at least two passages. Statistical significance was determined by unpaired Student's t-test or ANOVA, as appropriate. Differences were considered statistically significant when P<0.05.

## Results

### Intracellular pH following extracellular acidosis

**Figure 1:**
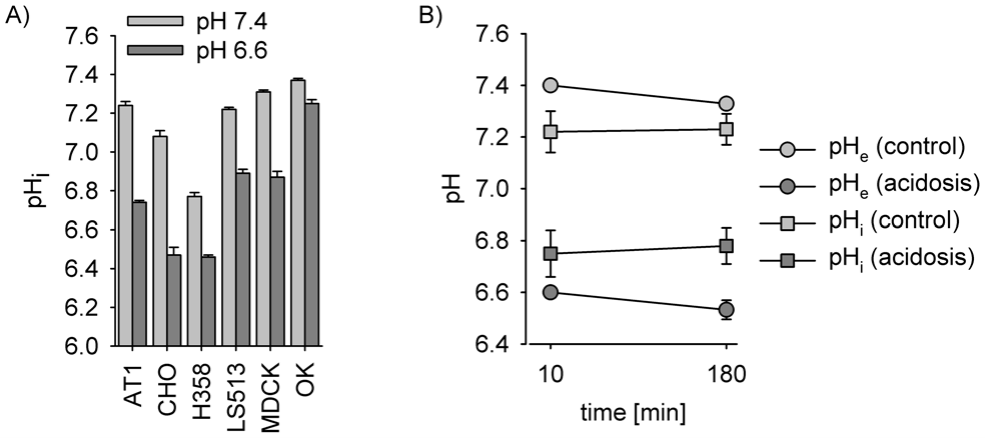
Effects of pH_e_ on pH_i_ in different cell lines. (A) pH_i_ measured under control conditions (pH 7.4) and during extracellular acidosis (pH 6.6) (N = 3–7). (B) pH-gradient in AT1 cells under control (pH 7.4) and acidic (pH 6.6) conditions for two different time points; circle: pH_e_, square: pH_i_.

Fig. 1A shows the intracellular pH of the different cell types. Lowering extracellular pH always led to a decrease in pH_i_, albeit the degree of this reduction differed strongly between the cell lines. Under control conditions (pH 7.4) the intracellular pH was always lower than in the extracellular compartment. However, under acidosis (pH 6.6) the intracellular pH was higher in most cell lines. This reverse pH-gradient has been described previously for solid tumors *in vivo* [6] indicating that the model used in the present study resembles the *in vivo* situation appropriately. Even though in CHO and H358 the intracellular pH has fallen below the extracellular pH there is indication for a pronounced net proton extrusion. From thermodynamic considerations in cells without proton extruders (assuming a membrane potential of −40 mV) the intracellular pH would be approximately 0.65 units lower as compared to the extracellular pH. In the experiments (pH_e_ 6.6) a pH_i_ of ∼6.0 would be expected from passive distribution. Obviously, CHO and H358 cells extrude protons actively against the passive equilibrium even though their capacity seems to be somewhat smaller than in the other cell lines.

The change of the intracellular pH rapidly follows the extracellular acidosis and was stable over several hours. For example, in AT1 cells intracellular pH (pH_i_) rapidly dropped from pH 7.22±0.08 to pH 6.75±0.09 within 10 min (Fig. 1B). This pH-change was sustained over 3 h (Fig. 1B), but reversible when switching back to a physiological pH_e_ (for regulation of pH-homeostasis in AT1 cells see Fig. S1). The decline in pH_e_ during 3 h incubation results from the extensive formation of lactic acid (Warburg effect, Fig. S2).

### Acidosis-induced MAPK activation in different cell types

**Figure 2:**
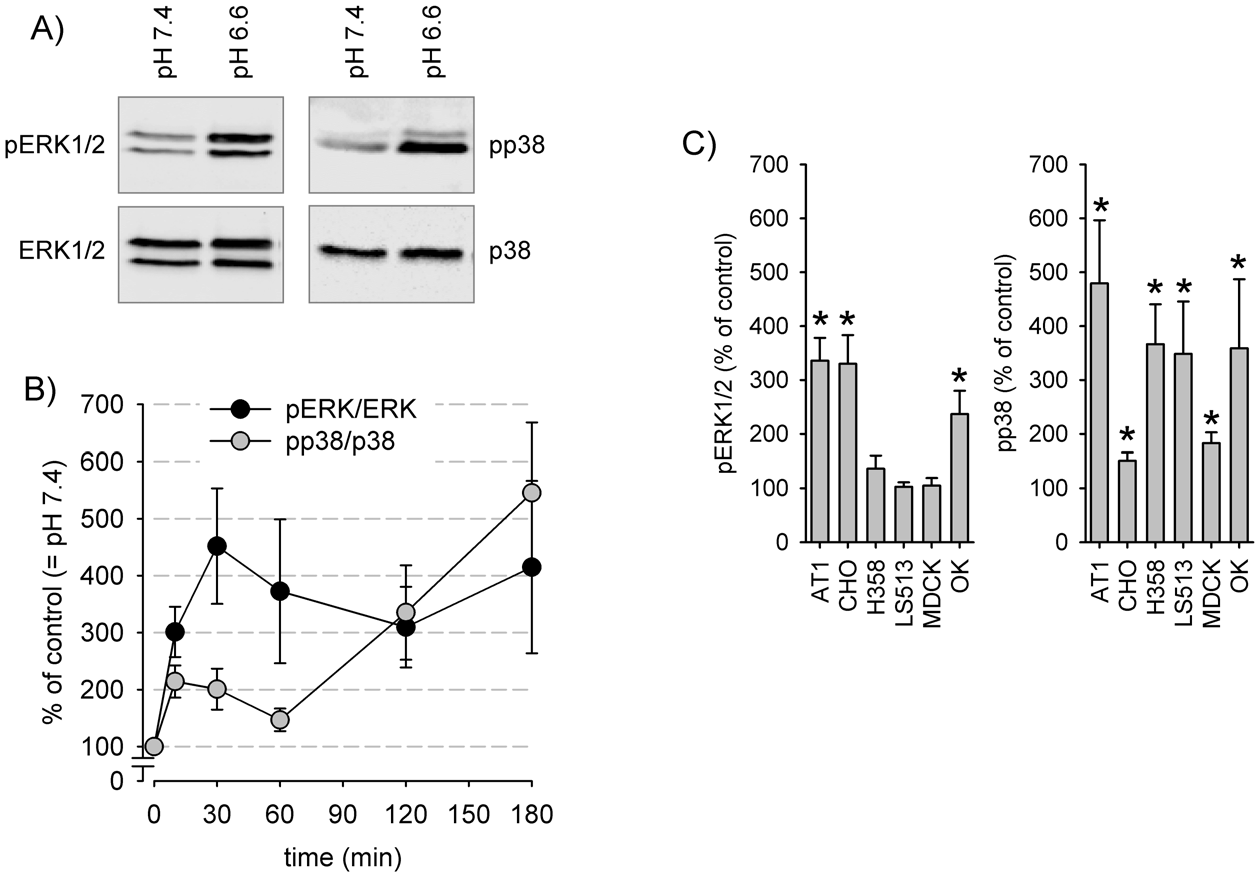
Dependency of MAPK phosphorylation on pH_e_ in different cell lines. (A) Typical western blot for phosphorylated and overall protein of ERK1/2 and p38 in AT1 cells after an incubation period of 3 h in either pH 7.4 or pH 6.6. (B) Time course of acidosis-induced ERK1/2 and p38 phosphorylation in AT1 cells. Values are phosphorylated protein divided by overall protein, 180 min HEPES-Ringer pH 7.4 is defined as 100%. (N = 3–10). (C) Impact of acidic conditions on ERK1/2 and p38 phosphorylation presented as % of control (pH 7.4) in various cell lines. (N = 5–12); (*) p<0.05.

Exposing cells to moderate extracellular acidosis leads to a marked activation of ERK1/2 and p38 MAP kinases. Fig. 2A shows the phosphorylation of both kinases after incubating AT1 cells for three hours at a pH of 6.6. Analyzing the time course shows that phosphorylation was already observed after 5 min and lasted up to 3 h (Fig. 2B). The effect on p38 activation was seen in a broad spectrum of various tumor or normal tissue cell lines (Fig. 2C). These findings indicate that p38 phosphorylation by acidosis is increased in different cells regardless if derived from tumor or normal tissue, pointing to a universal mechanism of stress-induced signaling. By contrast, acidosis-induced ERK1/2 activation was not observed in all cell types investigated and was not related to the tumorigenicity of the cells (Fig. 2C), although intracellular pH decreased in all cell lines. Thus, changes in pH_i_ seem not to be a universal trigger for acidosis-induced ERK1/2 phosphorylation. For both MAPK no quantitative correlation between pH_i_ or changes in intracellular pH and p38 or ERK1/2 phosphorylation for the different cell types was observed (Fig. S3). Taken together, these results led to the conclusion that bulk intracellular pH or changes in pH_i_ are not the key regulators for ERK1/2 phosphorylation induced by extracellular acidosis but serve as universal trigger for p38 phosphorylation. Since the principle mechanisms (intracellular acidification, p38 phosphorylation) were observed in AT1 cells further experiments were performed with this particular cell type.

### Effect of acidosis on cell viability

**Figure 3:**
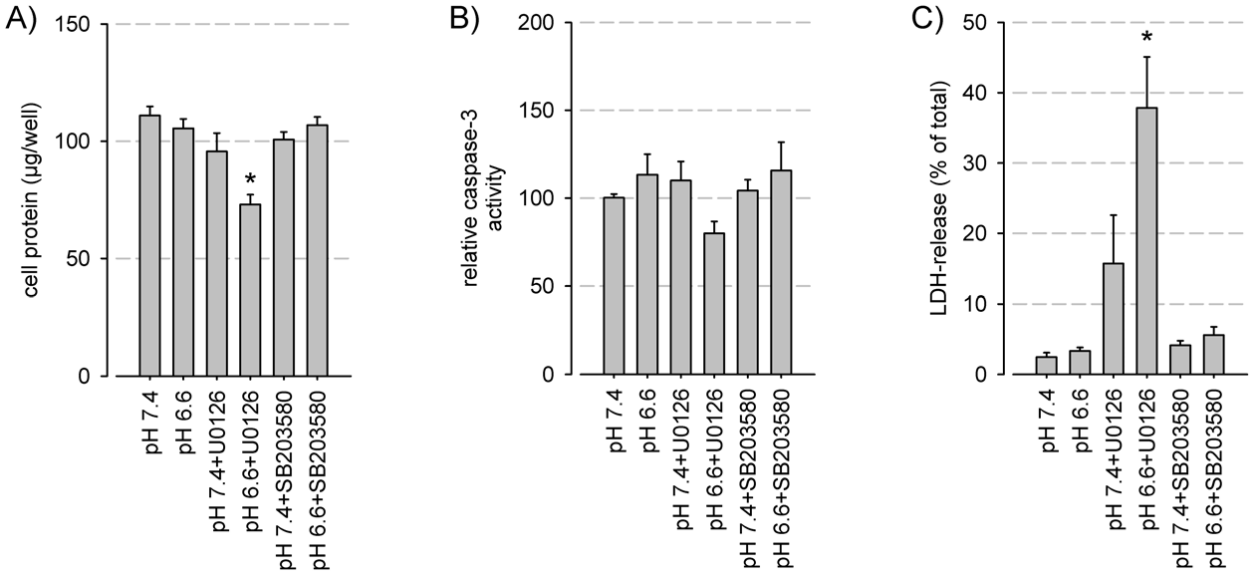
Impact of acidosis and MAPK-inhibitors on cell viability. (A) Cell protein content, (B) caspase-3-activity (apoptosis) and (C) LDH-release (necrosis) of AT1 cells after 3 h incubating at different pH with or without inhibitors of the ERK1/2 (U0126) or p38 (SB203580) pathway (N = 12–18); (*) p<0.05.

In order to assess different mechanisms of cell death the protein content per dish (overall cell viability), induction of apoptosis (caspase-3 activity) and necrosis (LDH-release) were analyzed. Acidosis per se elicited no significant change in cell survival (Fig. 3). Yet, when the ERK/2 pathway was inhibited necrotic cell death rose significantly, leading to a loss of cells. Caspase-3 activity was not affected, suggesting no alteration of apoptosis rate. Inhibition of the p38 pathway was virtually without effect. These data indicate that acidosis-induced ERK1/2 phosphorylation activates a rescue program preventing necrotic cell death. At present, the complete set of experiments in this series has only been performed in AT1 cells. For this reason it remains open whether the steps of acidosis-induced ERK1/2 activation followed by necrosis takes place in all cell lines as a general mechanism. Since some cell lines do not show an activation of ERK1/2 by acidosis (Fig. 2C) it might be possible that the ERK1/2-dependence of cell viability is at least quantitatively different among various cell lines. Here, further experiments are needed.

### Intracellular acidification and p38 phosphorylation

**Figure 4:**
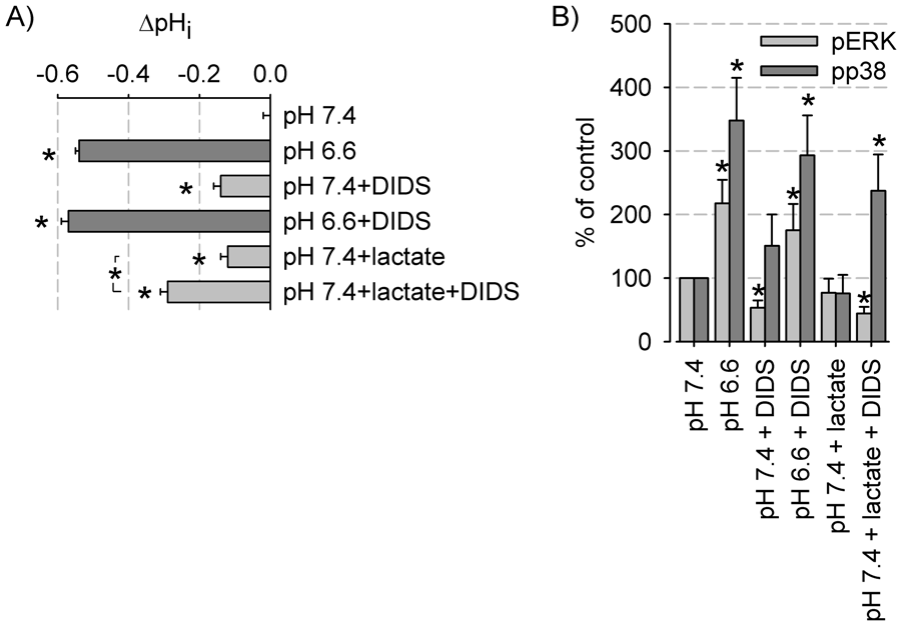
Impact of blocking bicarbonate transport on intracellular pH-regulation and MAPK phosphorylation. (A) Long term effects (3 h) of exposure to DIDS (200 µM) and/or addition of 40 mM lactate (N = 14–56) on pH_i_. pH-changes are compared to control conditions (pH 7.4) (*) p<0.05. (B) Ratio of phosphorylated MAPK/overall protein as % of control (pH 7.4); N = 3–7 for pERK; N = 4–9 for phospho-p38; (*) p<0.05.

The impact of intracellular acidification during inhibition of bicarbonate transport by DIDS and/or additional load with lactate on MAPK activation was studied. At a normal extracellular pH (7.4) either isolated inhibition of bicarbonate transport (200 µM DIDS) or incubation of the cells with lactate (40 mM) resulted in a rather weak acidification by 0.1 pH-units (Fig. 4A). However, the combination of both treatments decreased the pH markedly by 0.3 units. Analyzing MAPK activation under these conditions showed that sole inhibition of bicarbonate transport by DIDS or exposure of AT1 cells to lactate at physiological extracellular pH was not sufficient to alter p38 or ERK1/2 phosphorylation (Fig. 4B), most probably because pH_i_ recovers very rapidly in the presence of extracellular lactate (Fig. S1B). Simultaneous addition of DIDS and lactate resulted in significantly increased p38 phosphorylation (Fig. 4B). These data support the hypothesis that bulk changes in intracellular pH mediate the stimulatory effect of extracellular acidosis on p38 but not on ERK1/2. ERK1/2 phosphorylation is stimulated by a reduced extracellular pH but not by reduced intracellular pH (Fig. 4B).

### Acidosis-induced ERK1/2 and p38 phosphorylation is independent of phosphatase activity, but depends on MAPK kinases MEK1/2 and MKK3/6, respectively

**Figure 5:**
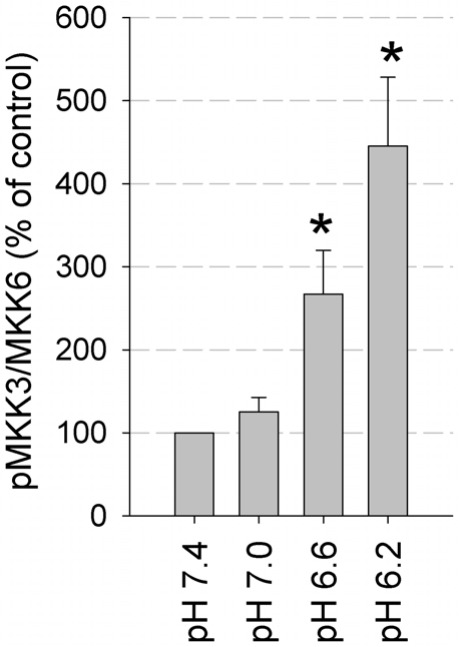
Acidosis-induced phosphorylation of MKK3/6. Values represent the ratio of phosphorylated protein (pp38 or pERK) divided by total protein (p38, ERK). These values have then been normalized to the ratio of control experiments at pH 7.4. (*) p<0.05 versus pH 7.4. (N = 4).

Since increased phosphorylation could be either the result of increased kinase activity or of reduced phosphatase rate the impact of phosphatase inhibition on the acidosis-induced MAPK activation was studied. When tyrosine- or theronine/serine-phosphatases were inhibited using either orthovanadate (100 µM) or ocadaic acid (100 nM) the effect of extracellular acidosis on ERK1/2 and p38 phosphorylation was additive (Fig. S4). In all experiments pp38 without phosphatase inhibitors was induced by the factor of 3-4 (Figs. S4 and S5) comparable to that found in previous experimental series (Fig. 2C). Thus, it is unlikely that the effect of acidosis is mediated by alterations of phosphatase activity. A key step in ERK1/2 activation is the upstream kinase MEK1/2, which integrates most of the signaling pathways converging on ERK1/2 [12]. When MEK1/2 was inhibited by 10 µM U0126, baseline phosphorylation of ERK1/2 was reduced substantially and the effect of acidosis was abrogated (Fig. S5). U0126 prevented acidosis-induced ERK1/2 phosphorylation even in the presence of the two phosphatase inhibitors orthovanadate and ocadaic acid, indicating that MEK1/2 is essential for the effect observed. p38 activation was not inhibited by U0126 (Fig. S5), but during 3 h acidosis (pH 6.6) the kinase MKK3/6, which is upstream of p38, was phosphorylated in a pH-dependent manner (Fig. 5). Taken together, our data suggest that acidosis-induced effects on MAPK do not depend on changes in phosphatase activity, whereas MEK1/2 is crucial for ERK1/2 activation and increased p38 phosphorylation is due to increased activity of MKK3/6 under acidic conditions.

### Acidosis-induced MAPK activation does not depend on EGFR, PKC, PKA, PI3K or Src family kinases

To unveil the possible signaling pathways leading from extracellular acidosis to an activation of ERK1/2 and p38, several promising targets were analyzed by blocking them with specific inhibitors. The role of epidermal growth factor receptor (EGFR), protein kinase C (PKC), protein kinase A (PKA), phosphatidylinositol 3-kinase (PI3K) and the Src family of tyrosine kinases was studied using AG1478, bisindolylmaleimide I (BIM), Rp-isomer, LY294002 and PP2, respectively. All of these kinases can play an important role in tumor promotion and progression. However, none of the inhibitors showed a significant effect on acidosis-induced ERK1/2 or p38 activation as depicted in Figs. S6 and S7, although maximal concentrations were used.

cAMP has a potential dual impact on MAPK signaling [12], [25]. Activation of PKA by cAMP can lead to inhibition of MEK1/2 and/or Raf-1 (C-Raf). However, activation of EPAC by cAMP activates B-Raf via Rap1. Application of the membrane permeable analogue dibutyryl-cAMP (db-cAMP, cAMP-clamp) *per se* led to reduced ERK1/2 but not p38 phosphorylation (Fig. S7). Additionally the effect of extracellular acidosis was completely abrogated. Stimulation of endogenous cAMP formation by forskolin (activator of adenylyl cyclases) elicited a similar effect as db-cAMP (Fig. S7). Thus, ERK1/2 but not p38 signaling is cAMP-sensitive and the effect is most probably mediated by PKA possibly via inhibition of C-Raf.

### OGR1 is not crucial for MAPK phosphorylation in an acidic microenvironment

In order to elucidate the mechanism of pH-sensing for MAPK activation further the role of G-protein-coupled receptors, able to sense extracellular protons/pH, has been studied. Of these, ovarian cancer G-protein-coupled receptor 1 (OGR1) has been shown to activate the MAPK pathway [26]. There are no specific inhibitors for OGR1 available, but it has been shown that proton-induced activation of ERK1/2 by OGR1 is abrogated by µM copper ion concentrations. Although this is no specific inhibition it can be used to falsify the hypothesis of OGR1 involvement. In the presence of 10 µM CuCl_2_ an increase in ERK1/2 and p38 phosphorylation was seen that was further enhanced by extracelluar acidosis (Fig. S8A). Thus, OGR1 does most probably not mediate the effect of extracellular acidosis on MAPK signaling.

### A role for Na^+^/K^+^-ATPase in acidosis-induced ERK1/2 activation?

Another possible mechanism for pH-"sensing" would be the Na^+^/K^+^-ATPase, an enzyme which is inhibited by acidosis and may induce MAPK activation either via alterations in cellular electrolyte homeostasis or by signaling via the EGFR [27], [28]. Fig. S8 shows that acidosis-induced inhibition of the Na^+^/K^+^-ATPase may contribute to ERK1/2 phosphorylation in AT1 cells. However, since cell volume changes which are part of the signaling cascade [29] were rather heterogeneous for the different cell lines, arguing against a decisive role for acidosis-induced ERK1/2 phosphorylation.

### Impact of ROS in the acidosis-induced MAPK activation

**Figure 6:**
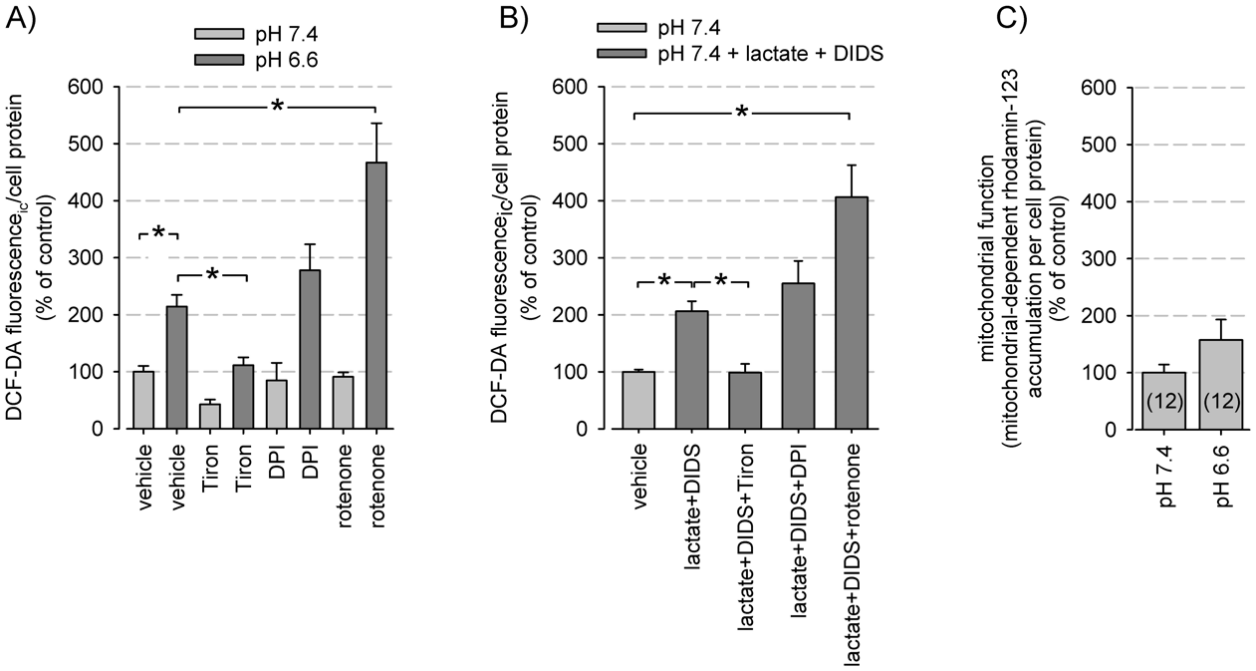
ROS formation induced by extra- or intracellular acidosis. (A) ROS formation during extracellular acidosis with application of the ROS scavengers tiron, DPI or rotenone (N = 9-12). (A) ROS formation (measured by DCF-DA fluorescence) during intracellular acidosis (induced by lactate+DIDS; see Fig. 4A) with application of the ROS scavengers tiron, DPI or rotenone (N = 3). (C) Relative mitochondrial activity (measured by rhodamine 123 accumulation) at control conditions (pH 7.4) and during extracellular acidosis (pH 6.6); N = 12. (*) p<0.05.

Since reactive oxygen species (ROS) can act as an intracellular signaling molecule [30], we analyzed whether ROS production changes during extracellular acidosis. Exposing cells to extracellular acidosis induced ROS formation (Fig. 6A). In order to distinguish between the impact of extra- and intracellular acidification cells were additionally incubated with lactate and DIDS at normal extracellular pH where the combination of both substances had the strongest impact on the intracellular pH (Fig. 4A) and elicited a significant increase in ROS formation (Fig. 6B). Scavenging ROS with tiron reduced the ROS level under control condition (pH 7.4) as well as during acidosis (Figs. 6A and B). DPI (diphenyleneiodonium chloride), an inhibitor of flavoproteins, such as NO synthase or NADPH oxidase, did not reduce ROS formation.

In the presence of rotenone, an inhibitor of mitochondrial complex I, ROS formation was enhanced under acidic but not under control conditions (Figs. 6A and B), i.e. acidosis stimulates rotenone-induced ROS formation. These results have been confirmed using the mitochondria-specific fluorescence probe MitoSox. With this probe mitochondrial ROS formation increased in the presence of rotenone to 236±44% at pH 7.4 but to 903±219% at pH 6.6 (n = 6; p<0.05) confirming a strong impact of acidic pH on ROS production in mitochondria. These data exclude mitochondrial reverse electron transport as a source of ROS but is indicative of an increased NADH/NAD^+^ ratio as trigger [31]. Complex I produces superoxide anion in the presence of NADH and this generation is enhanced by rotenone and ΔpH across the mitochondrial membrane. A gross activation of mitochondrial activity could not be detected (Fig. 6C).

**Figure 7:**
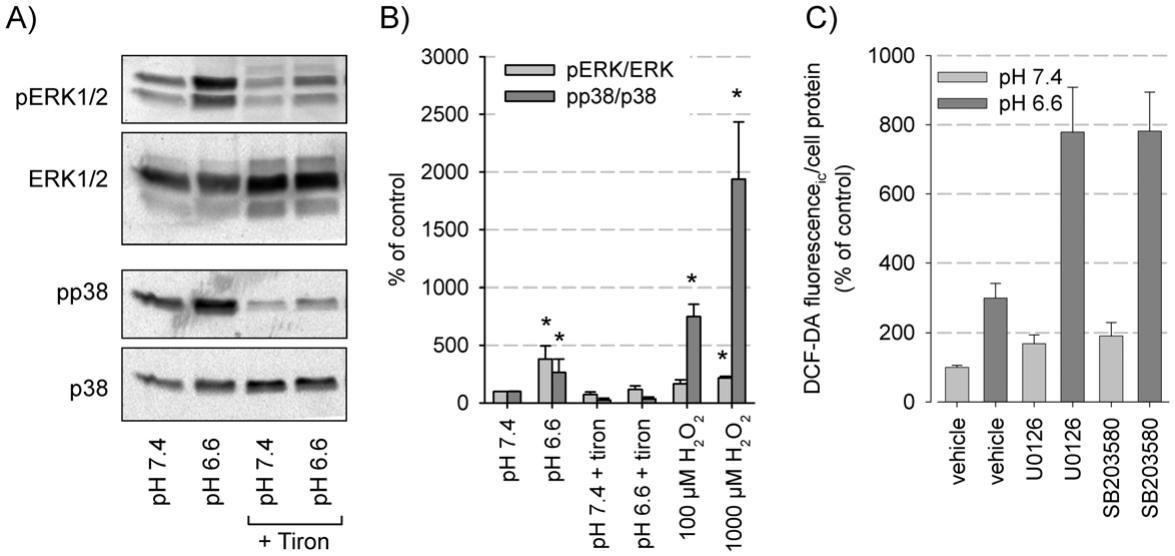
Impact of acidosis-induced ROS formation on MAPK activation. (A) Typical western blot for phosphorylated and overall protein of ERK1/2 and p38 in AT1 after an incubation period of 3 h in either pH 7.4 or pH 6.6 in the absence or presence of the ROS scavenger tiron. (B) Impact of ROS scavenging under acidic conditions or H_2_O_2_ incubation (at pH 7.4) on ERK1/2 and p38 phosphorylation. Values are normalized to control conditions at pH 7.4 (N = 6–7). (C) Effect of ERK1/2 (U0126) or p38 (SB203580) inhibition on ROS formation (N = 9–12). (*) p<0.05.

Incubating the cells with H_2_O_2_, a relatively stable ROS molecule, led to a dose dependent activation preferentially of the p38 MAP kinase (Fig. 7B). ERK1/2 phosphorylation was also increased. Incubating the cells with tiron which is able to markedly reduce ROS formation (Figs. 6A and B) inhibited both p38 and ERK1/2 activation (Figs. 7A and B), showing that ROS formation is upstream of MAPK phosphorylation. When the MAPK pathways were blocked (U0126 or SB203580) acidosis-induced ROS formation was enhanced (Fig. 7C), confirming that ROS are upstream of MAPK. Taken together, these results show that obviously the extra- as well as the intracellular acidosis induces ROS formation which, as a cause, then enhances MAPK phosphorylation.

### MAPK mediate acidosis-induced CREB activation

**Figure 8:**
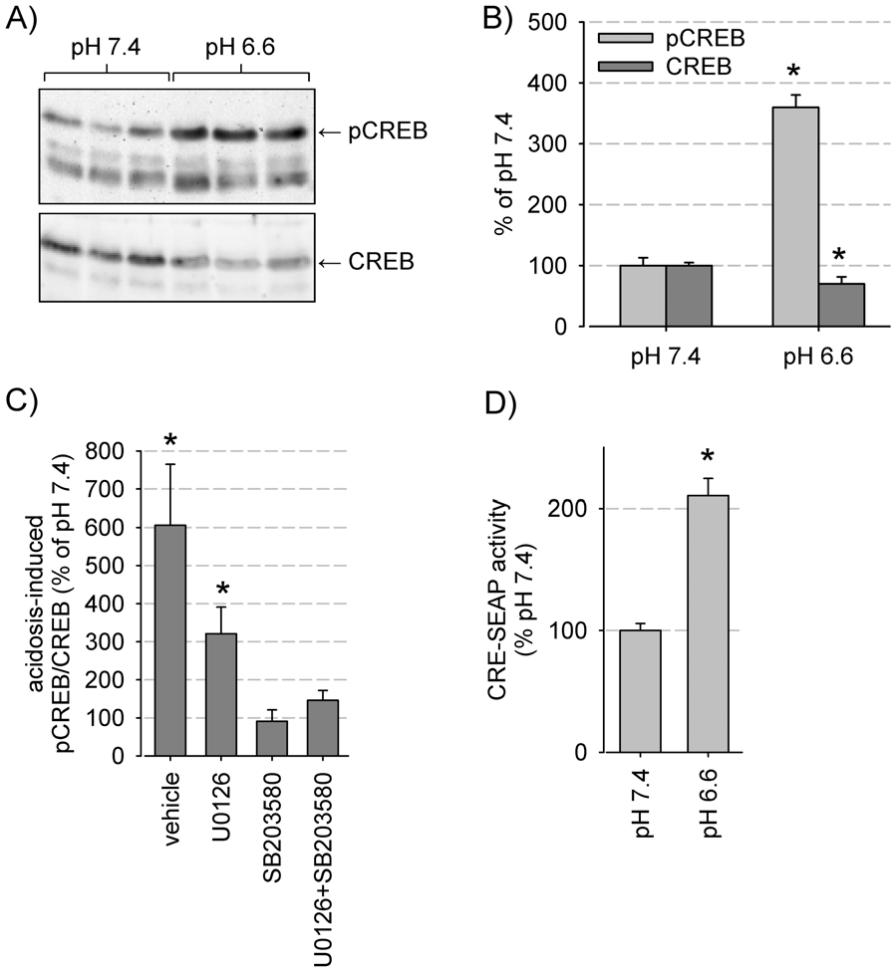
Impact of acidosis and MAPK activation on CREB phosphorylation and CRE activity. (A) Typical western blot for phosphorylated and overall protein of CREB in AT1 after an incubation period of 3h in either pH 7.4 or pH 6.6. (B) Impact of extracellular pH on total CREB and CREB phosphorylation. Measured pCREB and CREB levels were normalized to pH 7.4 (N = 8). The shown values describe the induction of expression compared to control values (pH 7.4) on the same blot. (C) Impact of inhibition of ERK1/2 (U0126) and/or p38 (SB203580) on acidosis-induced CREB phosphorylation (N = 3). Values shown describe the induction of CREB phosphorylation by pH 6.6 compared to that found at 7.4 in the absence or presence of MAPK inhibitors. Significance was calculated from the comparison of pCREB/CREB at pH 6.6+inhibitor with pH 7.4+inhibitor. (D) CRE activity (measured by the CRE-SEAP reporter gene assay) under control or acidic conditions (N = 20). (*) p<0.05.

MAPK may alter transcription factor activity, thereby affecting gene expression leading to long term effects. Among these transcription factors is CREB (CRE-binding protein), which has been shown to be involved in tumor initiation, progression and metastasis, supporting its role as a proto-oncogene [32]. It is well known that p38 activation can affect CREB phosphorylation and hence activity. In order to address the question whether an acidotic milieu may affect transcription by this pathway, phosphorylation of CREB as well as transcriptional CREB activity (by CRE reporter assay) was determined. Figs. 8A and B show that 3 h exposure to an acidotic environment leads to a marked CREB phosphorylation (Fig. 8C). CREB abundance was slightly decreased. Inhibition of the p38 pathway by the specific inhibitor SB203580 completely abrogated acidosis-induced CREB phosphorylation. Blocking the ERK1/2 pathway exerted no significant inhibition, although there was a tendency to lower phospho-CREB levels. Using a CRE-SEAP reporter gene assay [33] we observed that acidosis not only enhanced CREB phosphorylation but also CREB transcriptional activity (Fig. 8D). Of note, measurement of transcriptional activity started after a 3 h exposure period to acidosis and therefore represents an acidosis-induced effect persistent even when the cells were switched to control conditions (i.e. a “memory effect”).

## Discussion

### The model system

Inadequate perfusion in solid tumors leads to insufficient oxygen supply and forces the tumor cells to perform anaerobic glycolysis resulting in marked lactic acid formation and extracellular acidosis [6]. The cell system used in the present study represents a suitable model to study the crosstalk between tumor cell and microenvironment in cell culture, because (a) there is ample lactic acid production and (b) AT1 cells establish a reverse pH-gradient (pH_i_>pH_e_) during extracellular acidosis as compared to control conditions, similar to the situation described for tumor cells before [6]. Thus, under the experimental conditions of the present study the situation is qualitatively consistent with *in vivo* studies. Quantitatively, pH_i_ *in vivo* seems to be higher as compared to the situation in culture at similar extracellular pH-values. This apparent quantitative discrepancy has been observed before and might be due to a combination of the much larger extracellular space present in cell culture settings and the high buffer capacity of the medium representing a larger pool of protons that the cells can not compensate for [34].

### Acidosis-induced ERK1/2 and p38 phosphorylation

In all cell lines studied extracellular acidosis induced p38 phosphorylation within a few minutes which was sustained for the entire observation period whereas ERK1/2 phosphorylation occurred only in 3 of 6 cell types. Because acidosis induced a drop in pH_i_ and enhanced p38 phosphorylation in all cell lines tested, the link pH_i_→p38 seems to be of general validity. By contrast, ERK1/2 phosphorylation was not induced in all cell types tested, indicating that the link pH_e_→ERK1/2 is cell type-specific.

We observed no significant correlation between changes in pH_i_ and the degree of p38 phosphorylation in the different cell lines, indicating that the sensitivity of the pH_i_→p38 link is cell type-specific. Still, this does not exclude the existence of some kind of cell-specific threshold as suggested by [35], considering that intracellular acidification of AT1 cells using lactate alone was not sufficient to stimulate p38 phosphorylation whereas amplified intracellular acidification using lactate and DIDS was. These data also show that intracellular acidification activates p38 independently from changes in extracellular pH, a situation that may prevail in the initial phase of hypoxia [35]. The pH-sensitivity of ERK1/2 phosphorylation seems to be lower.

Our data concur with results from cardiomyocytes [35], also showing the pH_i_→p38 link. However, in contrast to cardiomyocytes, ERK1/2 phosphorylation required additional extracellular acidosis in AT1 cells, although we cannot exclude the possibility that ERK1/2 is less sensitive to pH changes because extracellular acidosis induced a larger drop in pH_i_ as compared to DIDS+lactic acid. Acidosis-induced p38 phosphorylation is most probably mediated by MKK3/6, because p38 itself is not pH-sensitive as shown by [35]. In AT1 cells acidosis also induced an approximately 3-fold induction of MKK3/6 phosphorylation. Not surprisingly, acidosis-induced ERK1/2 phosphorylation requires MEK1/2.

In principle, the question may arise whether serum starvation for 24 h (as used in our experiments) might be responsible for the activation of MAPK and for inducing autophagy. However, in all control experiments where the cells were kept at pH 7.4 comparable experimental conditions were used for the experimental groups and all samples were kept without serum for 24 h. For this reason an impact of serum depletion seems to be unlikely. Additional experiments show that in cells without serum starvation a comparable acidosis-induced MAPK activation can be observed (Fig. S9). Finally, the MAPK activation was seen within 5 min after change of pH (Fig. 2B), also indicating that serum starvation is not responsible for the observed effect.

In addition, autophagy has been linked with ROS induction, MAPK phosphorylation and cell death [36], [37]. However, in the present experiments acidosis per se was not able to reduce cell viability and no signs of apotosis, necrosis or loss of protein was seen (Fig. 3). Only with ERK1/2 inhibition necrotic cell death was induced (LDH release) resulting directly in a reduced cell mass, but no apoptosis was observed. These results indicate that induction of autophagy is unlikely for the actual results observed.

### Mechanisms involved in acidosis-induced MAPK phosphorylation

In order to unmask pathways involved in environmental acidosis-induced MAPK activation the impact of different signaling cascades was analyzed. We did not obtain any indication for the involvement of phosphatase activity, EGFR, PKC, PKA, PI3K or Src pathways. Furthermore, our data suggest that membrane proton receptors, namely OGR1 [26], do not contribute either. Of course, we are aware that conclusions drawn from pharmacological inhibitor studies have their limitations and need to be interpreted with care.

Another possible “signaling” mechanism that could be involved is cell volume, which may change under acidic conditions and modify the phosphorylation of a variety of proteins [38] such as tyrosine kinases, protein kinase C, adenylate cyclase, WNK and several mitogen activated protein kinases. However, the cell volume changes observed in this study varied profoundly between the cell types studied and did not correlate with the MAP kinase activation pattern, excluding volume changes as the underlying transduction mechanism.

Acidosis-induced changes in Na^+^/K^+^-ATPase activity could affect ERK1/2 phosphorylation via changes of intracellular sodium and subsequently calcium. Changes in the concentration of intracellular calcium in AT1 cells following acidosis have been described before [11]. However, the cytosolic calcium concentration decreased during acidosis making a calcium-driven mechanism unlikely. Beside alterations of Na^+^/K^+^-ATPase's activity, the pump can also serve as a signaling module in combination with EGFR, stimulating the ERK1/2 pathway [28]. However, these effects depend on activation of Src as well as transactivation of EGFR, both of which seem not involved in acidosis-induced ERK1/2 activation under our experimental conditions.

### The role of ROS

**Figure 9:**
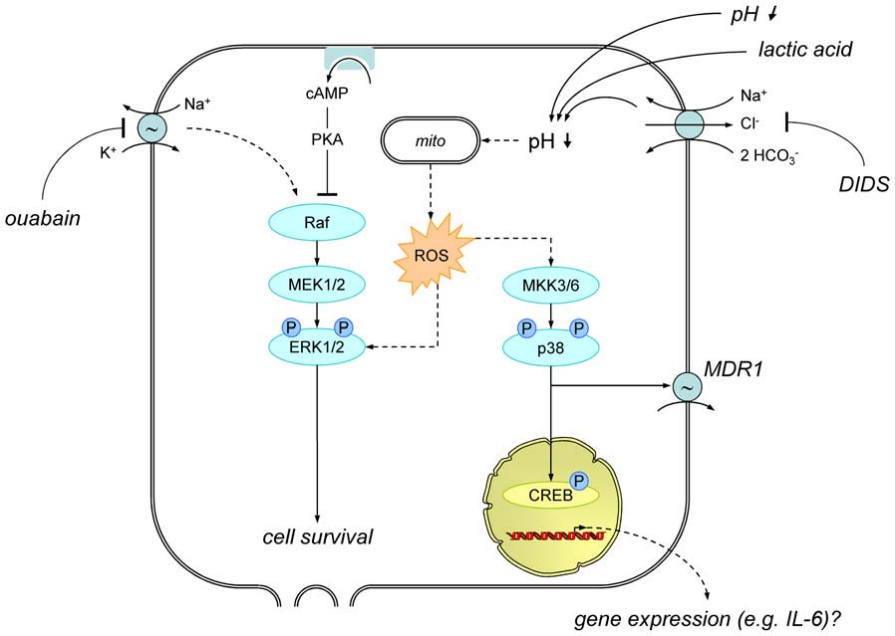
Model of the mechanism by which an acidic microenvironment affects ERK1/2 and p38 phosphorylation and the effects on cellular phenotype.

The results of the present study indicate that reactive oxygen species play a role in acidosis-induced p38 and ERK1/2 phosphorylation (Fig. 9). Scavenging of ROS with tiron prevented MAPK phosphorylation and direct application of ROS (H_2_O_2_) enhanced MAPK phosphorylation. These results are in good agreement with previous studies clearly demonstrating that the p38 pathway can be activated by ROS generated e.g. in complex I of the respiratory chain [39].

The question arises how the acidotic environment enhances ROS formation in tumor cells. ROS production was markedly stimulated by acidification (either extra- or intracellular) whereas DPI was not able to counteract this effect. These data argue against the involvement of NADPH-oxidase, NO-synthase or other flavo-proteins. Even though it is difficult to identify the source of ROS the most probable source in our case definitively are mitochondria. Here ROS will be generated at complex I when (i) mitochondrial ATP production is reduced (e.g., due to reduced ATP consumption or reduced aerobic glycolysis) resulting in a high proton gradient and a reduced CoQ (coenzyme Q) pool and when (ii) the NADH/NAD^+^ ratio is high [31]. ROS generation by reverse electron transfer in complex I is highly unlikely because it is inhibited by rotenone or DPI. However, we observed an increase of ROS formation by rotenone under acidic conditions. Rotenone-induced ROS formation is characteristic for complex I as the responsible source in the presence of a high NADH/NAD^+^ ratio, when respiration is low. An increase of mitochondrial activity under acidic conditions was not observed. Although the mechanism by which acidosis affects mitochondria still awaits final elucidation, mitochondria are the most probable source of oxygen radicals. The precise mechanisms have to be addressed in future studies.

### Conclusion

Acidosis-induced changes in MAPK activity are of potential importance for the tumor cell phenotype. Recently, we demonstrated that metabolic acidosis enhances chemoresistance in AT1 cells via p38 kinase [9]. Elias and Dias described the importance of p38 for acidosis-induced alternative splicing of VEGF [40]. ERK1/2 phosphorylation seems to serve as a rescue program limiting necrotic cell death during milieu acidosis. However, the underlying mechanisms have to be further investigated.

Finally, the present study shows that acidosis affects transcriptional activity through CREB phosphorylation. The transcription factor CREB has been shown to be involved in tumor initiation, progression and metastasis, supporting its role as a proto-oncogene [32], also for prostate cancer. Down-regulation of CREB in cancer cell lines results in inhibition of cell proliferation and induction of apoptosis, suggesting that CREB is an important transcription factor for determining the phenotype of certain tumor cells. Our data indicate that changes in CREB activity cannot only be the result of genetic alterations but also result from micromilieu perturbations, i.e. the tumor micromilieu itself, through mainly p38 kinase. In these terms, CREB represents a link for tumor microenvironment and genomic changes of tumor cell phenotype. Interestingly, increased CREB transcriptional activity was observed even 24 h after the cells had been switched back from the acidic milieu to pH_e_ 7.4. Thus, an acidic tumor microenvironment can induce a longer lasting change in the transcriptional program, representing a memory effect which maintains the altered phenotype even when the cells leave the tumor environment. Of course, these interpretations need future evaluation *in vivo*. Furthermore, we will have to investigate the identity of genes up-regulated by CREB under these conditions and whether there are other transcription factors affected.

## Supporting Information

Figure S1 **Regulation of the intracellular pH (pH_i_) in AT1 cells.** (A) Representative time course of pH_i_ after rapid changes of pH_e_ (n = 14). (B–D) Intracellular acidification and pH-recovery following the treatment with lactic acid. The accumulation of metabolically generated acids, as well as the movement of protons into the cell or bases out of the cell, constitutes a potentially deleterious acid challenge to the cell. This chronic acid load in tumor cells (to a great extend lactic acid formation) is overcome mainly by Na^+^/H^+^ antiport, Na^+^-dependent HCO_3_^−^/Cl^−^ exchange or possibly Na^+^-HCO_3_^−^ cotransport (i.e. by acid extruders). To characterize these processes in AT1 cells, which also show extensive lactic acid production (supplementary Fig. 2), and in order to determine the necessary experimental conditions for isolated intracellular acidosis, we studied the pH-recovery after an acid load using lactic acid (40 mM, pH_e_ 7.4). In the absence of any inhibitor, addition of lactic acid led to a rapid decrease (within a minute) in pH_i_ to pH 7.02 followed by a recovery within less than 7 min, as shown in (B; n = 14) and (C; n = 14–65). (D) Inhibition of Na^+^/H^+^ exchanger by 10 µM EIPA did not show a significant effect, whereas blocking bicarbonate transporters with 200 µM DIDS abrogated the pH-recovery after intracellular acidification (supplementary Fig. 1D). Recovery of pH_i_ per minute (see also B), n = 28–38. (*) p<0.05. This leads to the conclusion that bicarbonate transporters are crucial for pH-regulation in AT1 cells. This assumption is also supported by the following observations: (i) intracellular pH did not stay stable but decreased constantly when cells were superfused with solutions lacking bicarbonate, (ii) exposure to DIDS also led to a constant decrease in pH_i_ (E: pH_i_ time course after inhibiting DIDS-sensititve transporters; n = 14) and (iii) acute reduction of [Cl^−^]_e_ to 20 mM induced a prompt and reversible alkalinization (F: pH_i_ time course after reversible reduction of extracellular chloride; n = 14), suggesting that a Cl^−^-dependent transporter is involved. Because the driving forces for the Cl^−^/HCO_3_^−^-exchanger allow Cl^−^ entry and bicarbonate export at the prevailing pH-gradient ("acid loader"), this transporter cannot explain the data obtained (i.e. acidification during inhibition by DIDS). The present data suggest the existence of a Na^+^-dependent HCO_3_^−^/Cl^−^ exchange (NDCBE; Boron WF, Chen L, Parker MD. Modular structure of sodium-coupled bicarbonate transporters. J Exp Biol 2009 Jun 1;212(11):1697-706). In summary, exposure to lactate+DIDS generates isolated intracellular acidosis at normal pH_e_.
(PDF)

Figure S2 **Glycolysis-induced alteration of media parameters.** "H^+^-production" (A and B) as well as changes in the concentration of glucose (C) and lactate (D) within 3 h under control (pH 7.4) and acidic (pH 6.6) conditions (in 1 ml Ringer solution) were determined. AT1 cells were incubated with Ringer solutions for 3h and parameters were determined as mentioned. N = 10. The cell number (1.9±0.1 million cells per petri dish) did not differ significantly between the groups and was not affected during the three hour incubation period. Therefore the concentrations can be compared directly. (*) p<0.05.
(PDF)

Figure S3 **Acidosis-induced MAPK activation in different cell types.** There is no significant correlation between changes in pH_i_ or absolute pH_i_ and ERK1/2 or p38 phosphorylation in different cell types.
(PDF)

Figure S4 **Phosphatases do not mediate the effect of acidosis.** Inhibition of phosphatases by ocadaic acid (100 nM) or orthovanadate (100 µM) enhanced ERK1/2 and p38 phosphorylation per se. Acidosis exerted an additive effect on phosphorylation. (*) p<0.05. N = 4–6.
(PDF)

Figure S5 **Role of MEK1/2 in ERK1/2 phosphorylation.** Typical western blots and semi-quantitative analysis of the western blot data (N = 3) are shown. Values represent the ratio of phosphorylated protein (pp38 or pERK) divided by total protein (p38, ERK). These values have then been normalized to the ratio of control experiments at pH 7.4. OV, 100 µM orthovanadate; OA, 100 nM ocadaic acid; 10 µM U0126. (*) p<0.05.
(PDF)

Figure S6 **Effect of kinase inhibitors on acidosis-induced MAPK phosphorylation.** 1 µM AG1478 (EGFR kinase), 1 µM PP2 (Src-kinas), 1 µM BIM (protein kinase C), 10 µM Ly294002 (PI3-kinase). Values represent phosphorylated protein divided by total protein and normalized to HEPES-HCO_3_^−^-Ringer pH 7.4. (*) p<0.05 versus pH 7.4. N = 5–8.
(PDF)

Figure S7 **The role of cAMP in MAPK activation.** Addition of cAMP (300 µM db-cAMP) or stimualtion of cAMP formation (3 µM forskolin) prevented ERK1/2 but not p38 phosphorylation. The inactive cAMP analogue Rp-Isomer (300 µM) did not affect ERK1/2 or p38 phosphorylation. (*) p<0.05 versus pH 7.4. N = 5–7.
(PDF)

Figure S8 **The role of H^+^-sensors, cell volume and Na^+^/K^+^-ATPase in MAPK activation.** (A) Inhibtion of the H^+^-receptor ORG by 10 µM Cu^2+^ did not prevent acidosis-induced p38 or ERK1/2 phosphorylation. Representative of three blots. (B–D) Another possible mechanism for pH-"sensing" would be the Na^+^/K^+^-ATPase which is a pH-sensitive enzyme, inhibited by acidosis, that may induce MAPK activation either via alterations in cellular electrolyte homeostasis or by signaling via the EGFR. Exposing AT1 cells to an acidic extracellular environment showed a constant decrease in cell volume (B) which could be either the result of a reduced activity of the Na^+^/K^+^-ATPase or a lower Na^+^-transport via the above mentioned Na^+^-dependent HCO_3_^−^/Cl^−^ exchanger. Inhibiting the Na^+^/K^+^-ATPase by ouabain (100 µM) led to a reduction of the cell volume from 2093±82 fl to 1732±124 fl (p<0.05, N = 8) which was comparable to that found under acidic conditions (pH 6.6) 1839±65 fl (panel D; p<0.05, N = 8). As shown in (C), ouabain induced the phosphorylation of ERK1/2 but not of p38. Furthermore, the effect of extracellular acidosis on ERK1/2 but not on p38 was abrogated by ouabain. These data indicate that acidosis-induced inhibition of the Na^+^/K^+^-ATPase may contribute to ERK1/2 phosphorylation in AT1 cells. From these data it seems possible that cell shrinkage is part of the signaling cascade leading to ERK1/2 activation. However, cell volume changes were rather heterogeneous for the different cell lines (D), arguing against a decisive role for acidosis-induced ERK1/2 phosphorylation; (*) p<0.05 versus pH 7.4. N = 5
(PDF)

Figure S9 **ERK1/2 phosphorylation in AT1 cells growing without serum starvation.** In cells that were not serum deprived acidosis induced a similar ERK1/2 phosphorylation as compared to serum deprived cells (compare figure 2A).
(PDF)
