# Supplementary material for: Acidic Environment Leads to ROS-Induced MAPK Signaling in Cancer Cells
Source: PLoS One. 2011 Jul 26;6(7):e22445. doi: 10.1371/journal.pone.0022445 (PMC3144229; doi:10.1371/journal.pone.0022445)
Supplement: Figure S1 — Regulation of the intracellular pH (pHi) in AT1 cells. (A) Representative time course of pHi after rapid changes of pHe (n = 14). (B–D) Intracellular acidification and pH-recovery following the treatment with lactic acid. The accumulation of metabolically generated acids, as well as the movement of protons into the cell or bases out of the cell, constitutes a potentially deleterious acid challenge to the cell. This chronic acid load in tumor cells (to a great extend lactic acid formation) is overcome mainly by Na+/H+ antiport, Na+-dependent HCO3 −/Cl− exchange or possibly Na+-HCO3 − cotransport (i.e. by acid extruders). To characterize these processes in AT1 cells, which also show extensive lactic acid production (supplementary Fig. 2), and in order to determine the necessary experimental conditions for isolated intracellular acidosis, we studied the pH-recovery after an acid load using lactic acid (40 mM, pHe 7.4). In the absence of any inhibitor, addition of lactic acid led to a rapid decrease (within a minute) in pHi to pH 7.02 followed by a recovery within less than 7 min, as shown in (B; n = 14) and (C; n = 14–65). (D) Inhibition of Na+/H+ exchanger by 10 µM EIPA did not show a significant effect, whereas blocking bicarbonate transporters with 200 µM DIDS abrogated the pH-recovery after intracellular acidification (supplementary Fig. 1D). Recovery of pHi per minute (see also B), n = 28–38. (*) p<0.05. This leads to the conclusion that bicarbonate transporters are crucial for pH-regulation in AT1 cells. This assumption is also supported by the following observations: (i) intracellular pH did not stay stable but decreased constantly when cells were superfused with solutions lacking bicarbonate, (ii) exposure to DIDS also led to a constant decrease in pHi (E: pHi time course after inhibiting DIDS-sensititve transporters; n = 14) and (iii) acute reduction of [Cl−]e to 20 mM induced a prompt and reversible alkalinization (F: pHi time course after reversible reductio [file pone.0022445.s001.pdf]

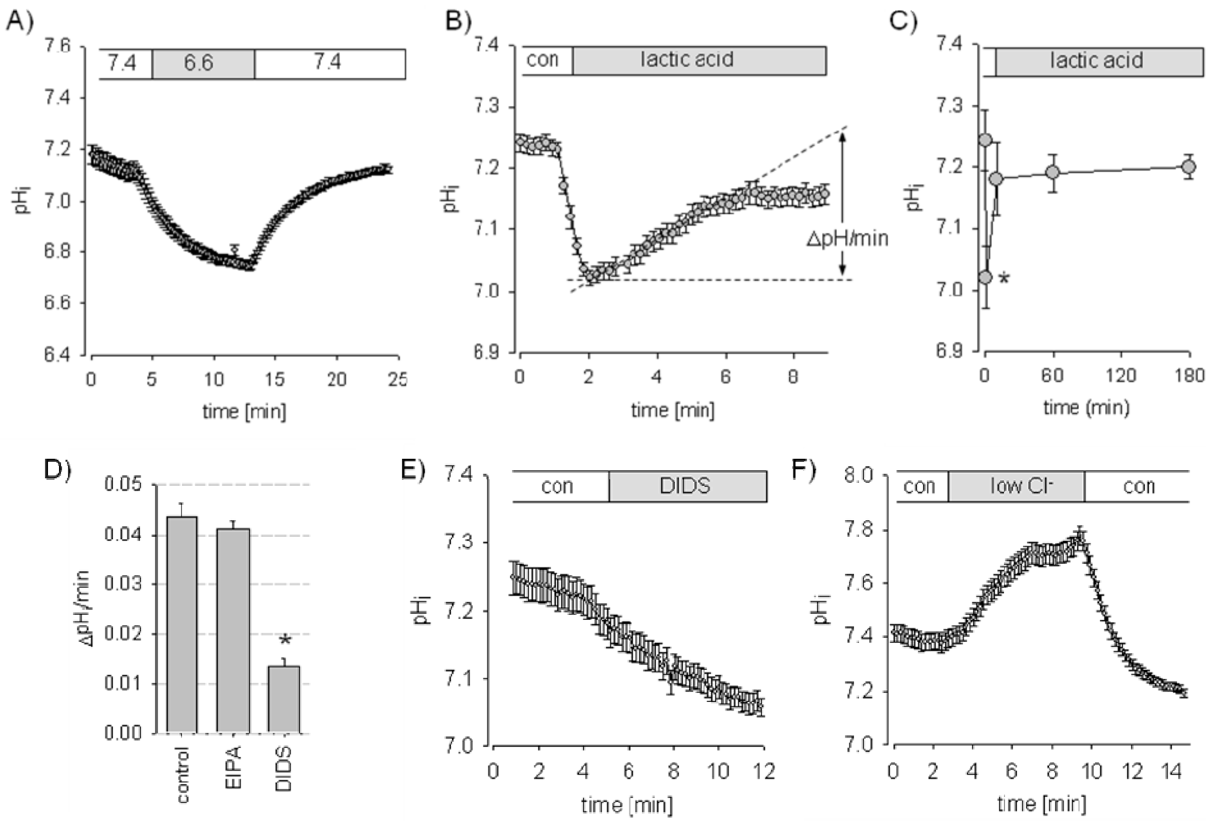

Figure S1. Regulation of the intracellular pH (pH_i) in AT1 cells. (A) Representative time course of pH_i after rapid changes of pH_e (n=14). (B-D) Intracellular acidification and pH-recovery following the treatment with lactic acid. The accumulation of metabolically generated acids, as well as the movement of protons into the cell or bases out of the cell, constitutes a potentially deleterious acid challenge to the cell. This chronic acid load in tumor cells (to a great extend lactic acid formation) is overcome mainly by Na^+/H^+ antiport, Na^+-dependent HCO_3^-/Cl^- exchange or possibly Na^+-HCO_3^- cotransport (i.e. by acid extruders). To characterize these processes in AT1 cells, which also show extensive lactic acid production (supplementary Fig. 2), and in order to determine the necessary experimental conditions for isolated intracellular acidosis, we studied the pH-recovery after an acid load using lactic acid (40 mM, pH_e 7.4). In the absence of any inhibitor, addition of lactic acid led to a rapid decrease (within a minute) in pH_i to pH 7.02 followed by a recovery within less than 7 min, as shown in (B; n=14) and (C; n=14-65). (D) Inhibition of Na^+/H^+ exchanger by 10 µM EIPA did not show a significant effect, whereas blocking bicarbonate transporters with 200 µM DIDS abrogated the pH-recovery after intracellular acidification (supplementary Fig. 1D). Recovery of pH_i per minute (see also B), n=28-38. (*) $p<0.05$. This leads to the conclusion that bicarbonate transporters are crucial for pH-regulation in AT1 cells. This assumption is also supported by the following observations: (i) intracellular pH did not stay stable but decreased constantly when cells were superfused with solutions lacking bicarbonate, (ii) exposure to DIDS also led to a constant decrease in pH_i (E: pH_i time course after inhibiting DIDS-sensititve transporters; n=14) and (iii) acute reduction of $[Cl^-]_e$ to 20 mM induced a prompt and reversible alkalinization (F: pH_i time course after reversible reduction of extracellular chloride; n=14), suggesting that a Cl^--dependent transporter is involved. Because the driving forces for the Cl^-/HCO_3^--exchanger allow Cl^- entry and bicarbonate export at the prevailing pH-gradient ("acid loader"), this transporter cannot explain the data obtained (i.e. acidification during inhibition by DIDS). The present data suggest the existence of a Na^+-dependent HCO_3^-/Cl^- exchange (NDCBE; Boron WF, Chen L, Parker MD. Modular structure

of sodium-coupled bicarbonate transporters. J Exp Biol 2009 Jun 1;212(11):1697-706). In summary, exposure to lactate+DIDS generates isolated intracellular acidosis at normal pH_e.
