# Supplementary material for: Acidic Environment Leads to ROS-Induced MAPK Signaling in Cancer Cells
Source: PLoS One. 2011 Jul 26;6(7):e22445. doi: 10.1371/journal.pone.0022445 (PMC3144229; doi:10.1371/journal.pone.0022445)
Supplement: Figure S2 — Glycolysis-induced alteration of media parameters. "H+-production" (A and B) as well as changes in the concentration of glucose (C) and lactate (D) within 3 h under control (pH 7.4) and acidic (pH 6.6) conditions (in 1 ml Ringer solution) were determined. AT1 cells were incubated with Ringer solutions for 3h and parameters were determined as mentioned. N = 10. The cell number (1.9±0.1 million cells per petri dish) did not differ significantly between the groups and was not affected during the three hour incubation period. Therefore the concentrations can be compared directly. (*) p<0.05. (PDF) [file pone.0022445.s002.pdf]

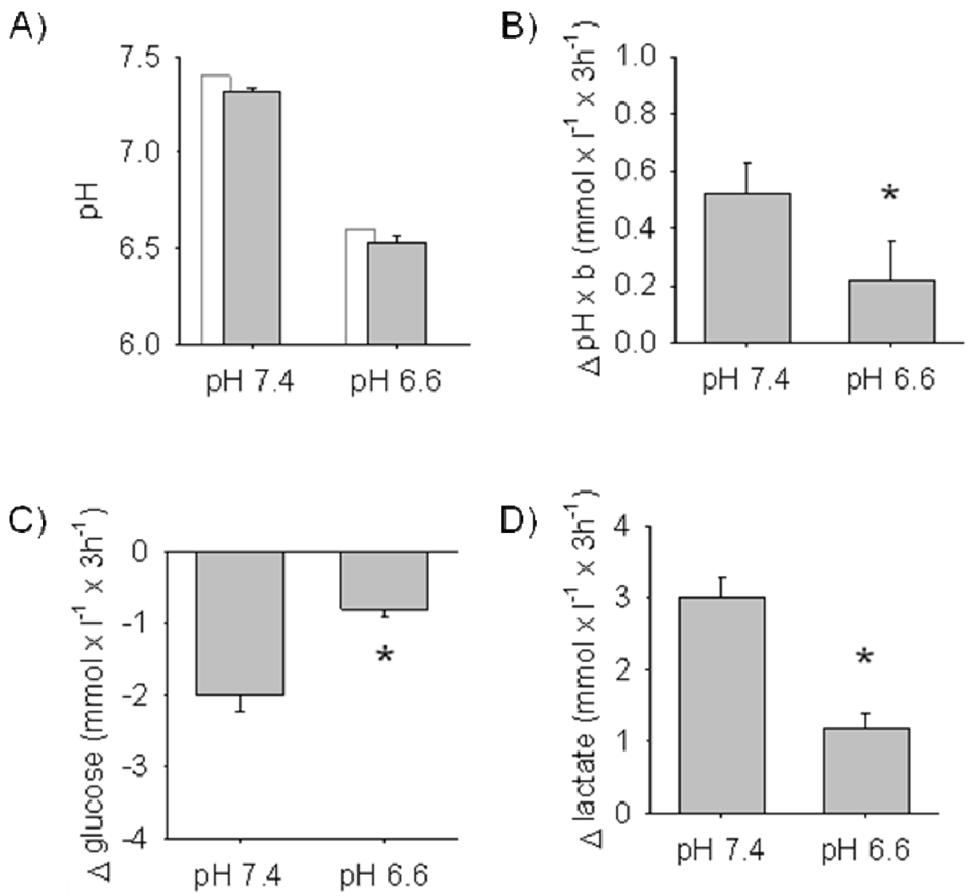

Figure S2. Glycolysis-induced alteration of media parameters. "H^+-production" (A and B) as well as changes in the concentration of glucose (C) and lactate (D) within 3 h under control (pH 7.4) and acidic (pH 6.6) conditions (in 1 ml Ringer solution) were determined. AT1 cells were incubated with Ringer solutions for 3h and parameters were determined as mentioned. N = 10. The cell number (1.9±0.1 million cells per petri dish) did not differ significantly between the groups and was not affected during the three hour incubation period. Therefore the concentrations can be compared directly. (*) $p<0.05$.
