# Supplementary material for: Acidic Environment Leads to ROS-Induced MAPK Signaling in Cancer Cells
Source: PLoS One. 2011 Jul 26;6(7):e22445. doi: 10.1371/journal.pone.0022445 (PMC3144229; doi:10.1371/journal.pone.0022445)
Supplement: Figure S3 — Acidosis-induced MAPK activation in different cell types. There is no significant correlation between changes in pHi or absolute pHi and ERK1/2 or p38 phosphorylation in different cell types. (PDF) [file pone.0022445.s003.pdf]

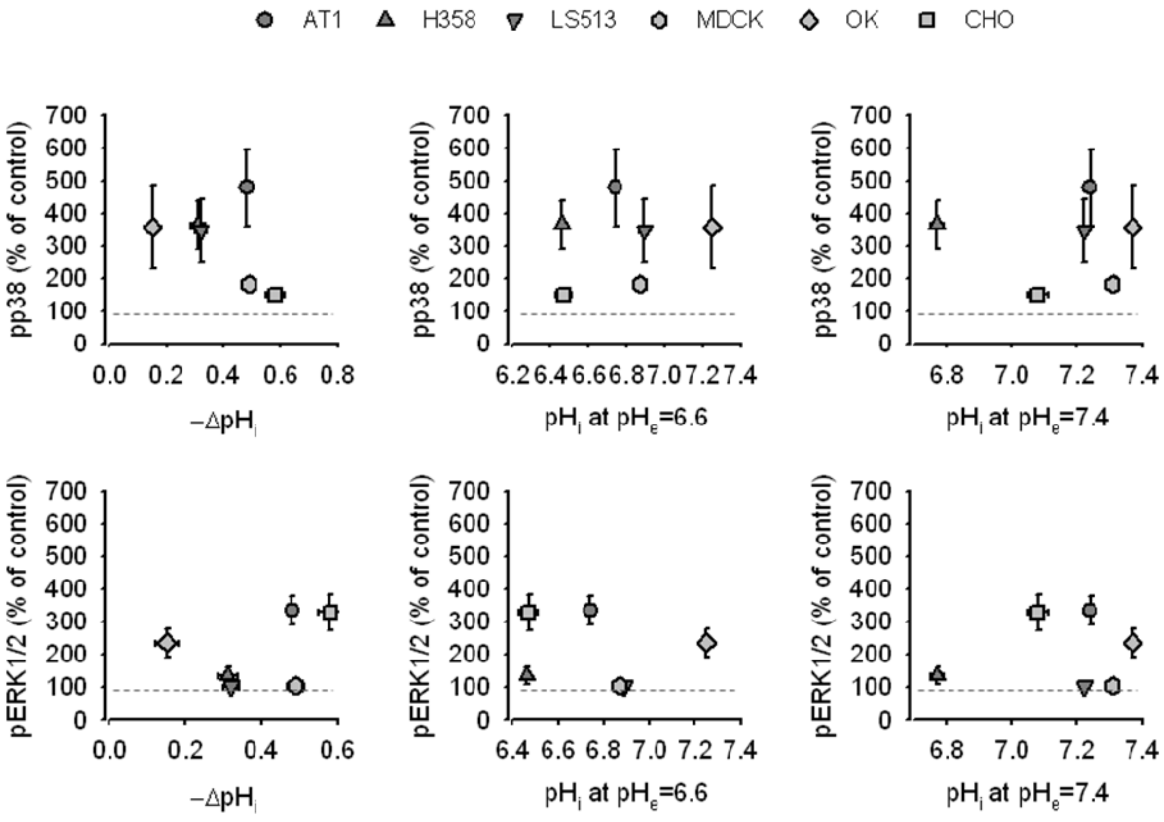

Figure S3. Acidosis-induced MAPK activation in different cell types. No significant correlation between changes in pH_i or absolute pH_i and ERK1/2 or p38 phosphorylation in different cell types.
