# Supplementary material for: Acidic Environment Leads to ROS-Induced MAPK Signaling in Cancer Cells
Source: PLoS One. 2011 Jul 26;6(7):e22445. doi: 10.1371/journal.pone.0022445 (PMC3144229; doi:10.1371/journal.pone.0022445)
Supplement: Figure S4 — Phosphatases do not mediate the effect of acidosis. Inhibition of phosphatases by ocadaic acid (100 nM) or orthovanadate (100 µM) enhanced ERK1/2 and p38 phosphorylation per se. Acidosis exerted an additive effect on phosphorylation. (*) p<0.05. N = 4–6. (PDF) [file pone.0022445.s004.pdf]

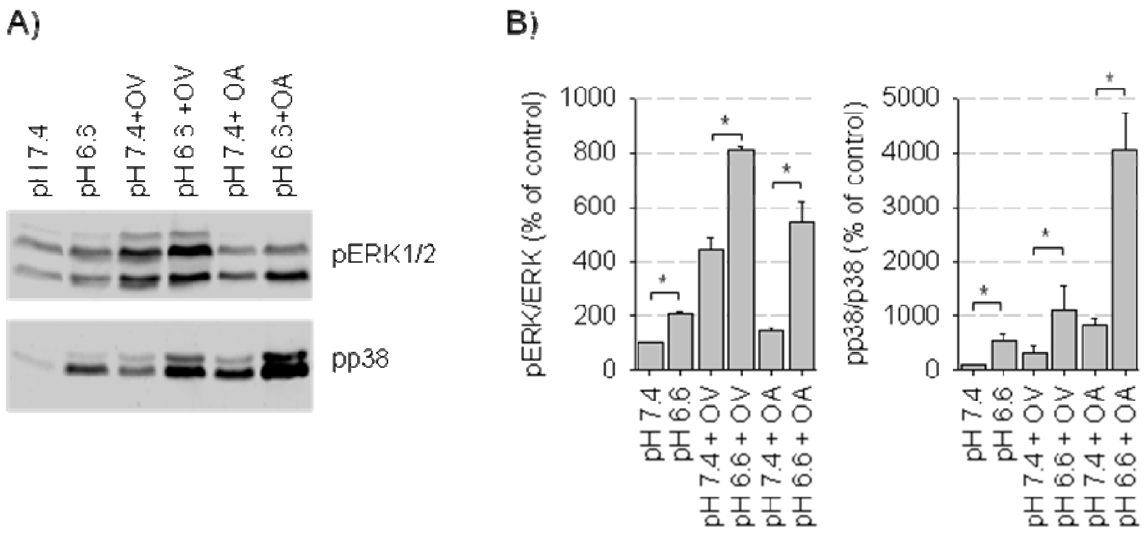

Figure S4. Phosphatases do not mediate the effect of acidosis. Inhibition of phosphatases by ocadaic acid (100 nM) or orthovanadate (100 µM) enhanced ERK1/2 and p38 phosphorylation per se. Acidosis exerted an additive effect on phosphorylation. (*) $p<0.05$. N= 4-6.
