# Supplementary material for: Acidic Environment Leads to ROS-Induced MAPK Signaling in Cancer Cells
Source: PLoS One. 2011 Jul 26;6(7):e22445. doi: 10.1371/journal.pone.0022445 (PMC3144229; doi:10.1371/journal.pone.0022445)
Supplement: Figure S5 — Role of MEK1/2 in ERK1/2 phosphorylation. Typical western blots and semi-quantitative analysis of the western blot data (N = 3) are shown. Values represent the ratio of phosphorylated protein (pp38 or pERK) divided by total protein (p38, ERK). These values have then been normalized to the ratio of control experiments at pH 7.4. OV, 100 µM orthovanadate; OA, 100 nM ocadaic acid; 10 µM U0126. (*) p<0.05. (PDF) [file pone.0022445.s005.pdf]

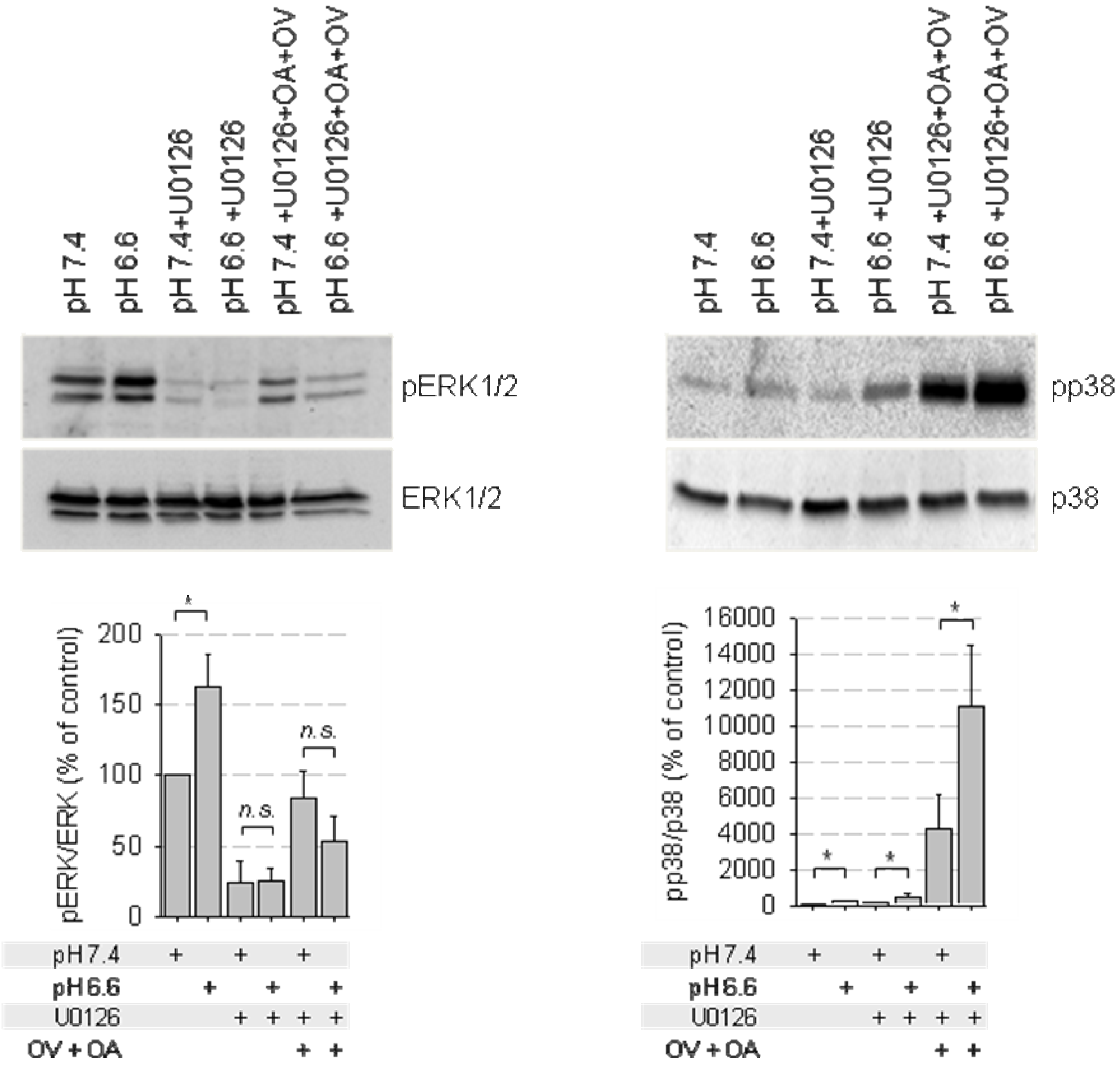

Figure S5. Role of MEK1/2 in ERK1/2 phosphorylation. Typical western blots and semi-quantitative analysis of the western blot data (N = 3) are shown. Values represent the ratio of phosphorylated protein (pp38 or pERK) divided by total protein (p38, ERK). These values have then been normalized to the ratio of control experiments at pH 7.4. OV, 100 µM orthovanadate; OA, 100 nM ocadaic acid; 10 µM U0126. (*) $p<0.05$.
