# Supplementary material for: Acidic Environment Leads to ROS-Induced MAPK Signaling in Cancer Cells
Source: PLoS One. 2011 Jul 26;6(7):e22445. doi: 10.1371/journal.pone.0022445 (PMC3144229; doi:10.1371/journal.pone.0022445)
Supplement: Figure S6 — Effect of kinase inhibitors on acidosis-induced MAPK phosphorylation. 1 µM AG1478 (EGFR kinase), 1 µM PP2 (Src-kinas), 1 µM BIM (protein kinase C), 10 µM Ly294002 (PI3-kinase). Values represent phosphorylated protein divided by total protein and normalized to HEPES-HCO3 −-Ringer pH 7.4. (*) p<0.05 versus pH 7.4. N = 5–8. (PDF) [file pone.0022445.s006.pdf]

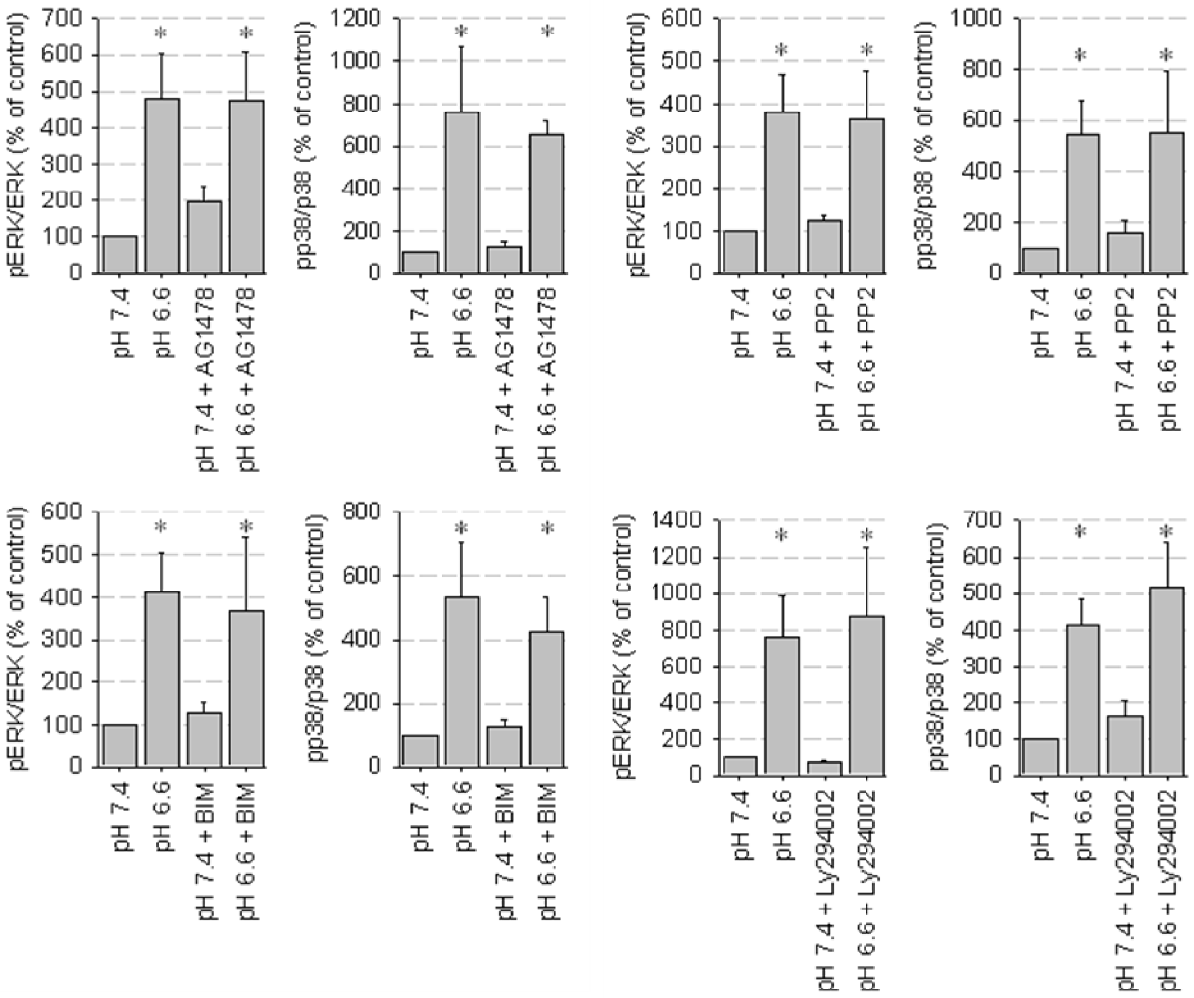

Figure S6. Effect of kinase inhibitors on acidosis-induced MAPK phosphorylation. 1 µM AG1478 (EGFR kinase), 1 µM PP2 (Src-kinas), 1 µM BIM (protein kinase C), 10 µM Ly294002 (PI3-kinase). Values represent phosphorylated protein divided by total protein and normalized to HEPES-HCO_3^--Ringer pH 7.4. (*) $p<0.05$ versus pH 7.4. N=5-8.
