# Supplementary material for: Acidic Environment Leads to ROS-Induced MAPK Signaling in Cancer Cells
Source: PLoS One. 2011 Jul 26;6(7):e22445. doi: 10.1371/journal.pone.0022445 (PMC3144229; doi:10.1371/journal.pone.0022445)
Supplement: Figure S7 — The role of cAMP in MAPK activation. Addition of cAMP (300 µM db-cAMP) or stimualtion of cAMP formation (3 µM forskolin) prevented ERK1/2 but not p38 phosphorylation. The inactive cAMP analogue Rp-Isomer (300 µM) did not affect ERK1/2 or p38 phosphorylation. (*) p<0.05 versus pH 7.4. N = 5–7. (PDF) [file pone.0022445.s007.pdf]

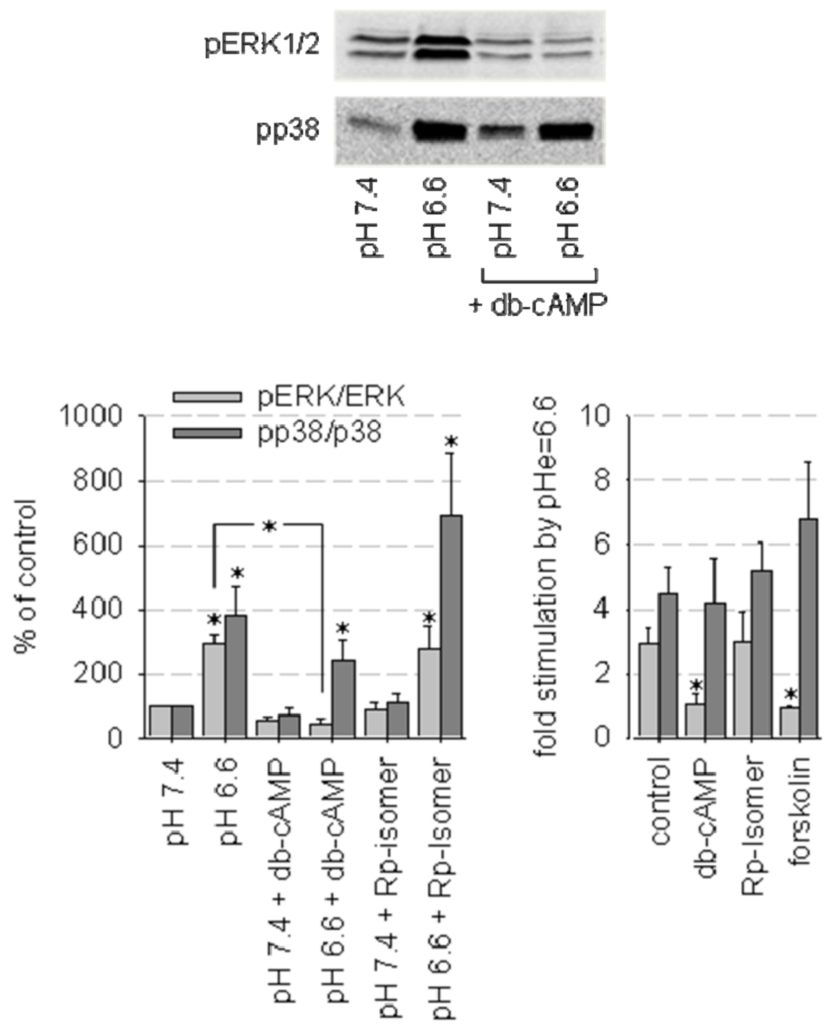

Figure S7. The role of cAMP in MAPK activation. Addition of cAMP (300 µM db-cAMP) or stimualtion of cAMP formation (3 µM forskolin) prevented ERK1/2 but not p38 phosphorylation. The inactive cAMP analogue Rp-Isomer (300 µM) did not affect ERK1/2 or p38 phosphorylation. (*) $p<0.05$ versus pH 7.4. N=5-7.
