# Supplementary material for: Acidic Environment Leads to ROS-Induced MAPK Signaling in Cancer Cells
Source: PLoS One. 2011 Jul 26;6(7):e22445. doi: 10.1371/journal.pone.0022445 (PMC3144229; doi:10.1371/journal.pone.0022445)
Supplement: Figure S8 — The role of H+-sensors, cell volume and Na+/K+-ATPase in MAPK activation. (A) Inhibtion of the H+-receptor ORG by 10 µM Cu2+ did not prevent acidosis-induced p38 or ERK1/2 phosphorylation. Representative of three blots. (B–D) Another possible mechanism for pH-"sensing" would be the Na+/K+-ATPase which is a pH-sensitive enzyme, inhibited by acidosis, that may induce MAPK activation either via alterations in cellular electrolyte homeostasis or by signaling via the EGFR. Exposing AT1 cells to an acidic extracellular environment showed a constant decrease in cell volume (B) which could be either the result of a reduced activity of the Na+/K+-ATPase or a lower Na+-transport via the above mentioned Na+-dependent HCO3 −/Cl− exchanger. Inhibiting the Na+/K+-ATPase by ouabain (100 µM) led to a reduction of the cell volume from 2093±82 fl to 1732±124 fl (p<0.05, N = 8) which was comparable to that found under acidic conditions (pH 6.6) 1839±65 fl (panel D; p<0.05, N = 8). As shown in (C), ouabain induced the phosphorylation of ERK1/2 but not of p38. Furthermore, the effect of extracellular acidosis on ERK1/2 but not on p38 was abrogated by ouabain. These data indicate that acidosis-induced inhibition of the Na+/K+-ATPase may contribute to ERK1/2 phosphorylation in AT1 cells. From these data it seems possible that cell shrinkage is part of the signaling cascade leading to ERK1/2 activation. However, cell volume changes were rather heterogeneous for the different cell lines (D), arguing against a decisive role for acidosis-induced ERK1/2 phosphorylation; (*) p<0.05 versus pH 7.4. N = 5 (PDF) [file pone.0022445.s008.pdf]

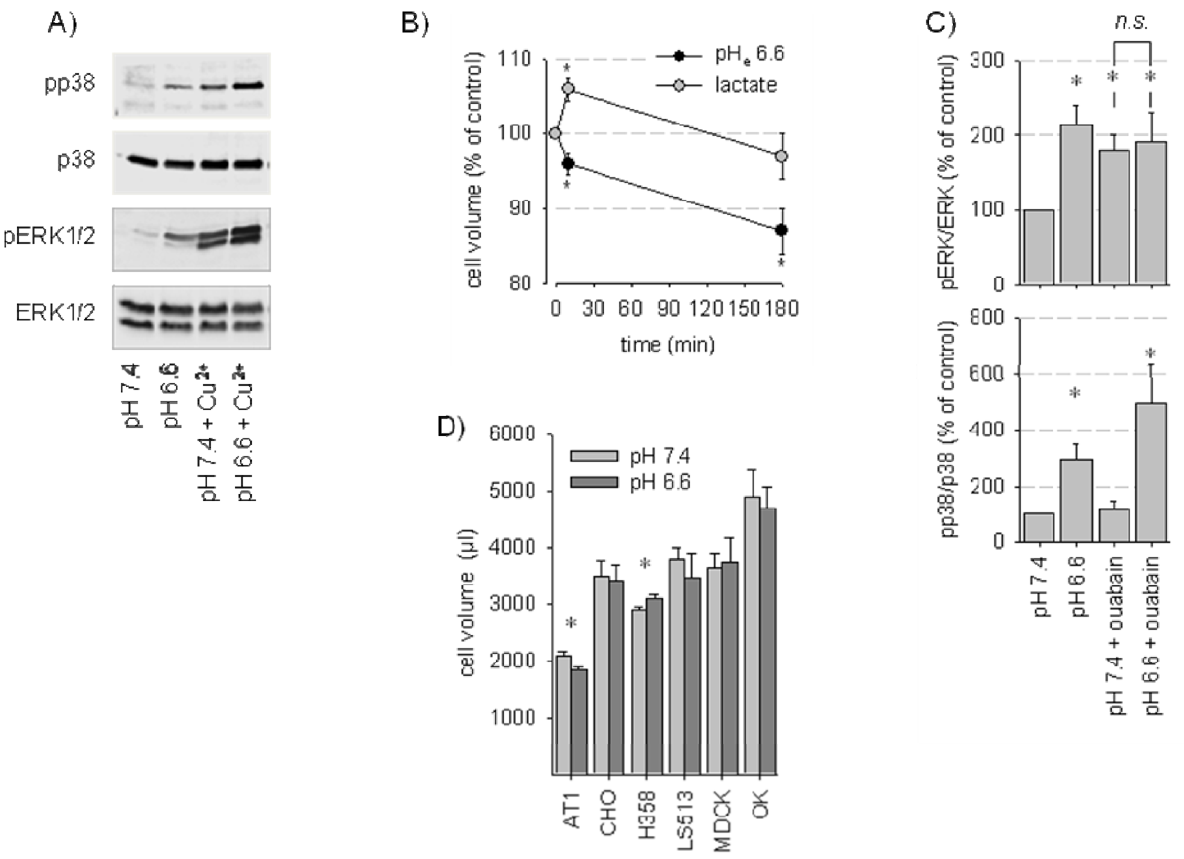

Figure S8. The role of H^+-sensors, cell volume and Na^+/K^+-ATPase in MAPK activation. (A) Inhibtion of the H^+-receptor ORG by 10 µM Cu^{2+} did not prevent acidosis-induced p38 or ERK1/2 phosphorylation. Representative of three blots. (B-D) Another possible mechanism for pH-"sensing" would be the Na^+/K^+-ATPase which is a pH-sensitive enzyme, inhibited by acidosis, that may induce MAPK activation either via alterations in cellular electrolyte homeostasis or by signaling via the EGFR. Exposing AT1 cells to an acidic extracellular environment showed a constant decrease in cell volume (B) which could be either the result of a reduced activity of the Na^+/K^+-ATPase or a lower Na^+-transport via the above mentioned Na^+-dependent HCO_3^-/Cl^- exchanger. Inhibiting the Na^+/K^+-ATPase by ouabain (100 µM) led to a reduction of the cell volume from 2093±82 fl to 1732±124 fl ($p<0.05$, N=8) which was comparable to that found under acidic conditions (pH 6.6) 1839±65 fl (panel D; $p<0.05$, N=8). As shown in (C), ouabain induced the phosphorylation of ERK1/2 but not of p38. Furthermore, the effect of extracellular acidosis on ERK1/2 but not on p38 was abrogated by ouabain. These data indicate that acidosis-induced inhibition of the Na^+/K^+-ATPase may contribute to ERK1/2 phosphorylation in AT1 cells. From these data it seems possible that cell shrinkage is part of the signaling cascade leading to ERK1/2 activation. However, cell volume changes were rather heterogeneous for the different cell lines (D), arguing against a decisive role for acidosis-induced ERK1/2 phosphorylation; (*) $p<0.05$ versus pH 7.4. N=5
