# Supplementary material for: Acidic Environment Leads to ROS-Induced MAPK Signaling in Cancer Cells
Source: PLoS One. 2011 Jul 26;6(7):e22445. doi: 10.1371/journal.pone.0022445 (PMC3144229; doi:10.1371/journal.pone.0022445)
Supplement: Figure S9 — ERK1/2 phosphorylation in AT1 cells growing without serum starvation. In cells that were not serum deprived acidosis induced a similar ERK1/2 phosphorylation as compared to serum deprived cells (compare figure 2A). (PDF) [file pone.0022445.s009.pdf]

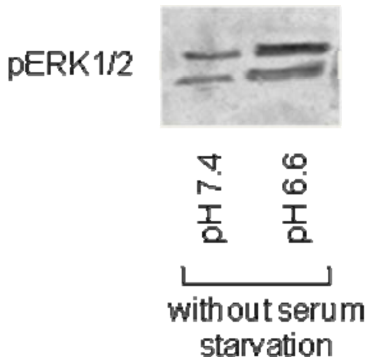

Figure S9. ERK1/2 phosphorylation in AT1 cells growing without serum starvation. In cells that were not serum deprived acidosis induced a similar ERK1/2 phosphorylation as compared to serum deprived cells (compare figure 2A).
